# Selection of Target Sites for Mobile DNA Integration in the Human Genome

**DOI:** 10.1371/journal.pcbi.0020157

**Published:** 2006-11-24

**Authors:** Charles Berry, Sridhar Hannenhalli, Jeremy Leipzig, Frederic D Bushman

**Affiliations:** 1 Department of Family and Preventive Medicine, School of Medicine, University of California San Diego, La Jolla, California, United States of America; 2 Department of Genetics, University of Pennsylvania School of Medicine, Philadelphia, Pennsylvania, United States of America; 3 Department of Microbiology, University of Pennsylvania School of Medicine, Philadelphia, Pennsylvania, United States of America; University of California Davis, United States of America

## Abstract

DNA sequences from retroviruses, retrotransposons, DNA transposons, and parvoviruses can all become integrated into the human genome. Accumulation of such sequences accounts for at least 40% of our genome today. These integrating elements are also of interest as gene-delivery vectors for human gene therapy. Here we present a comprehensive bioinformatic analysis of integration targeting by HIV, MLV, ASLV, SFV, L1, SB, and AAV. We used a mathematical method which allowed annotation of each base pair in the human genome for its likelihood of hosting an integration event by each type of element, taking advantage of more than 200 types of genomic annotation. This bioinformatic resource documents a wealth of new associations between genomic features and integration targeting. The study also revealed that the length of genomic intervals analyzed strongly affected the conclusions drawn—thus, answering the question “What genomic features affect integration?” requires carefully specifying the length scale of interest.

## Synopsis

Many types of genomic parasites insert their DNA sequences into the human genome. Among these are retroviruses such as HIV, transposons, and adeno-associated virus. These integrating elements are important for the changes they cause in the human genome upon insertion of new DNA, and as gene-delivery vehicles for use in gene therapy. Previous studies have generated sequences of genomic targets of integration by these elements. Here Berry, Hannenhalli, Leipzig, and Bushman present a comprehensive bioinformatic analysis that allows them to annotate each base pair in the human genome for its likelihood of hosting an integration event by each type of element. This resource should prove useful in understanding genomic evolution and optimizing gene delivery vectors for use in human gene therapy.

## Introduction

The exons of human genes comprise only about 1.5% of the total genome sequence [1,2]. Fragments of genomic parasites—integrating viruses and transposons—comprise a much larger fraction, at least 40%. These elements are also highly dynamic—new elements insert and occasionally excise, and repeated sequences provide portable regions of sequence homology that act as substrates for homologous recombination. Integration of new DNA can result in changes in gene activity or formation of new genes [3,4].

Integrating DNA elements are also important in human gene therapy as delivery vehicles for new sequences. Recent setbacks in gene therapy, however, emphasize the importance of integration target site selection. In an otherwise quite successful gene therapy trial treating human X-SCID, the gene therapy vector used integrated near a proto-oncogene and caused leukemia in three of the patients treated [5,6].

Here we present a detailed bioinformatic analysis of integration targeting in the human genome by seven types of integrating elements, taking advantage of the extensive sequence data available on de novo sites of integration [7]. For the retroviruses, published genome-wide surveys of integration target sites have shown that human immunodeficiency virus (HIV), murine leukemia virus (MLV), avian sarcoma–leukosis virus (ASLV), and simian foamy virus (SFV) all show different patterns of favored integration sites. HIV favors integration in active transcription units (TUs) [8–16], while MLV favored integration near gene 5′ ends [9,16]. ASLV shows the most random target site distribution, favoring TUs only slightly [10,17]. SFV integration sites are also relatively randomly distributed, though a favoring of integration near CpG islands could be detected [18].

Long interspersed nuclear elements (LINEs) are non-LTR retrotransposons that replicate via transcription, then reverse transcription primed by a nick in the genomic target DNA [3,4,19,20]. LINE-related sequences comprise fully 20% of the human DNA [1,2]. LINEs are the only known class of human transposons that are active for transposition, the others being inactive molecular fossils. Two previous surveys of integration targeting by engineered human L1 LINEs emphasized that the integration reaction often rearranges the target site DNA as a consequence of the coupled reverse transcription–integration mechanism [21,22]. The published studies reached different conclusions on whether or not TUs were favored integration targets.

Another class of transposons is exemplified by Sleeping Beauty (SB). This element transposes via a “cut-and-paste” mechanism, involving excision of the SB DNA from the genome and reintegration at a new site [3,4]. SB integration site selection in vivo has been studied by two groups, revealing that integration sites were nearly randomly distributed in the genome, showing only a weak favoring of TUs [23,24].

The last integrating element studied is the parvovirus adeno-associated virus (AAV). AAV does not integrate as a normal step in its life cycle, but under certain growth conditions a portion of the viral DNA can become integrated in the host cell chromosome. In infections with wild-type AAV, integration can take place preferentially at a specific locus on human Chromosome 19. However, in infections with AAV-based vectors that do not express the viral Rep protein, integration is not site-specific [25]. In the AAV-vector dataset studied here, integration was reported to take place with a modest preference for regions near transcription start sites [26,27]. Integration under these conditions is not carried out by an AAV-encoded integrase/transposase enzyme, but apparently by host enzymes involved in repair of DNA double-strand breaks. During the repair process, AAV sequences are proposed to become incorporated so as to bridge between the broken DNA ends [28].

Though integration takes place at many genomic locations, favored specific nucleotide sequences can be detected in the target DNA at the point of integration for most of these elements. For LINEs and SB, this nucleotide sequence is strongly conserved among sites [23,24,26,27,29,30]. For retroviruses, conservation is weaker but still significant [31–34]. In some cases, studies in vitro have shown that the favored target sequence is a property of the element-encoded integration enzymes. For example, a synthetic version of the favored HIV integration sequence 5′GT(A/T)AC3′ was shown to be a preferential integration target for HIV integration complexes in vitro [32]. Similar studies are available for MLV, ASLV, L1, and DNA transposons [3,35–38], but not for SFV and AAV. How much these favored sequences influence integration-site selection genome-wide has not been fully clarified.

Here we present a comprehensive statistical comparison of the factors influencing integration frequency by annotating each base pair in the human genome for its relative likelihood of hosting integration events. Combined effects of genomic features were then assessed, involving analysis of more than 200 variables over 17 integration-site datasets. These variables consist of recognizable genomic features such as gene density, CpG islands, DNase I cleavage sites, etc., analyzed over intervals of varying lengths, thereby resulting in >200 measures. To construct the combined model, Bayes model averaging and the machine learning algorithm RandomForest were used to sample the “model space” efficiently and thereby clarify effects due to correlation among variables (i.e., “confounding effects,” in statistical terminology).

The effects of interval sizes used for comparison were also assessed. For example, in trying to evaluate the potential hazards of gene therapy, one might want to know whether the promoter regions for certain oncogenes were particularly favorable integration targets compared with other promoters. We found that conclusions may be different and even opposite depending on the interval size studied.

In the Results section, the analysis is organized around each type of genomic annotation. The data are summarized as color-coded “heat maps,” allowing use of these findings as an encyclopedia for assessing the effects of genomic features on integration targeting by each element. In the Discussion section, new findings are presented in turn for each class of integrating element.

## Results

### Datasets Studied

The integration site collections studied are listed in Table 1, together with the original references. To generate each dataset, engineered elements were induced to carry out integration in cultured human cells. After allowing time for integration, genomic DNA was harvested, and human DNA flanking the integrated element was cloned and sequenced. For all of the elements except AAV it was possible to obtain integration site datasets from multiple cell types. Comparison among these datasets provides information on the influence of the cell type.

**Table 1:**
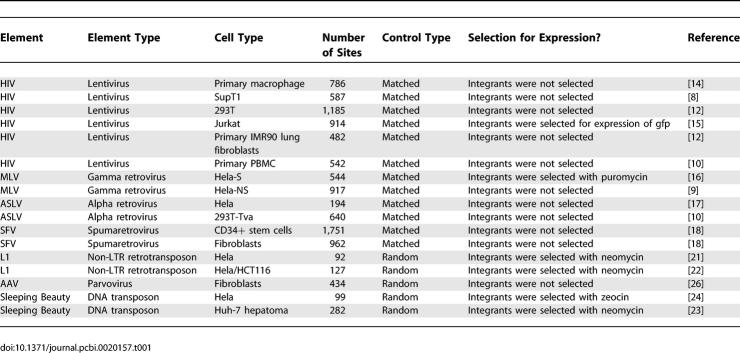
Integration Site Datasets Used in This Study

Another variable was the treatment of cells after infection. In some cases, recovery of the integration sites involved use of a selectable marker carried on the integrating element. As a result, the integrated element was only recovered if it supported gene expression. Previous work has suggested that selection for expression biased the recovery of integration sites [15,16], and this analysis is extended below.

For statistical analysis, the integration site datasets were compared with randomly selected control sites in the human genome. Many of the sites were cloned by methods involving use of restriction enzymes to cleave genomic DNA flanking integrated elements. Thus there arises a concern that the placement of restriction enzyme cleavage sites in the human genome could bias the recovery of integration sites. For many of the datasets studied, it was possible to correct for possible biases by using a matched random control, in which each experimentally generated integration site was paired with ten random sites in the human genome that were constrained to lie the same number of base pairs from an appropriate restriction site. In the statistical analysis, each experimental integration site was compared with its matched random controls, thereby controlling for possible bias from restriction enzyme cleavage. For a few integration site datasets, it was not practical to generate matched random controls, so unmatched random sites were used (Table 1).

For comparison of integration frequency to transcriptional activity, microarray data was used to annotate genes for their relative activity. In most cases it was possible to use array data from the cell types used in the integration study (Table S1).

### Associations of Genomic Features with Integration

Our statistical approach is summarized in the following sections. A more comprehensive treatment can be found in Text S1–S3.

**Figure 1:**
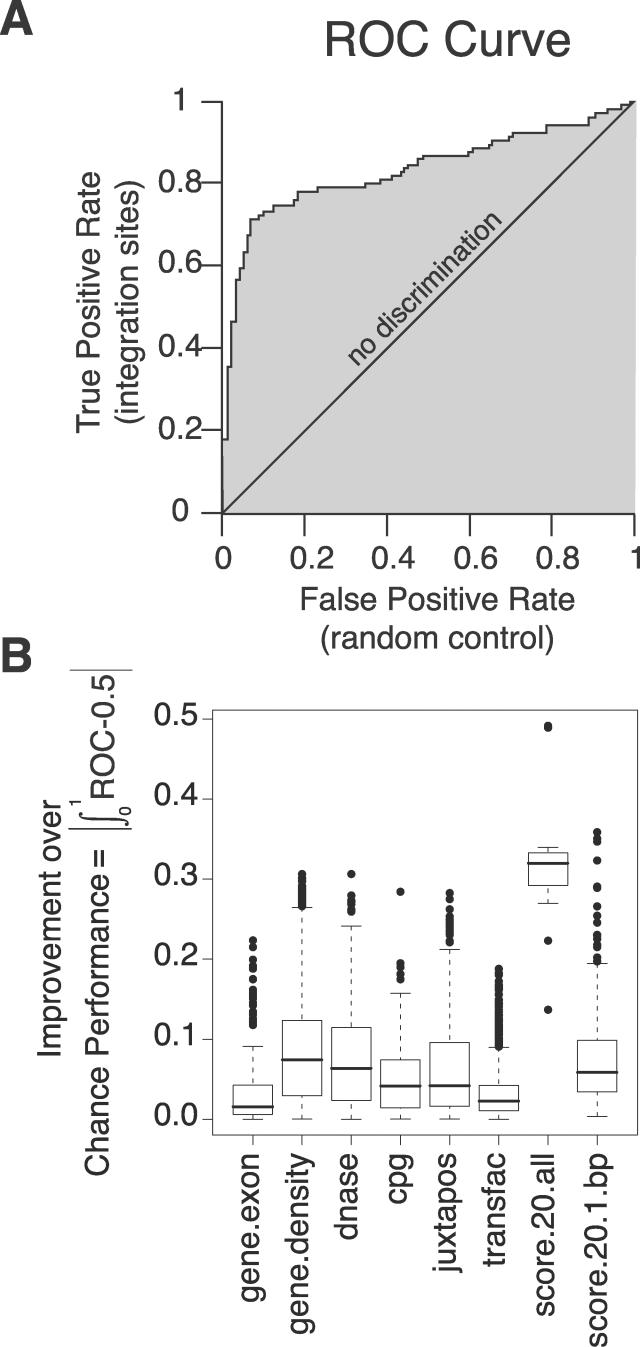
ROC Curves as a Measure of the Association of DNA Integration with Genomic Features (A) Diagram of the ROC analysis. The graph plots the true positive rate against the false positive rate for every possible cutpoint; vertical steps result when only the true positive rate increases as the cutpoint (i.e., cutoff value for the genomic feature) moves down; horizontal steps result when only the false positive rate increases, and when both rates increase as the cutpoint moves down the graph “steps” diagonally. The example shows the effects of score.20 on SB integration (though the method of construction is general). The area between the curve and the “no discrimination” line indicates discrimination between integration sites and random controls by the predictor tested. The curve will lie beneath the line of “no discrimination”—leading to an area of less than 0—if integration sites tend to have lower values of the variable under study than random controls. For details see the text and Text S1. (B) Box plots summarizing ROC results. Each box in Figure 1B indicates the first and third quartiles of the values, while the heavy line in the middle gives the median value. The “whiskers” extend to the most extreme observation within 1.5× the interquartile range of the median. Points that lie beyond the whiskers are plotted individually. For each box plot, the number of points is 17 (the number of datasets) times the number of rows in the relevant heat map for that feature (in Text S2; selected examples of heat maps are shown in Figures 2–4). Specifically, the numbers of points were 170 for gene.exon, 1173 for gene.density, 153 for dnase, 306 for cpg, 340 for juxtapos, 1870 for transfac, 17 for score.20.all, and 340 for score.20.1.bp.

The variables used describe characteristics of the genomic sequence surrounding the integration sites or controls (a detailed catalog of genomic features is in Text S2, pp. 3–4). To analyze effects of genomic features on integration, we used a common measure of a predictor variable's ability to discriminate between two classes of events, which is the area under the receiver operator characteristic (ROC) curve (for background on ROC curves, see [39]). An example of an ROC curve is presented in Figure 1A, and a detailed explanation is presented in Text S1. For the analysis, experimental and control integration sites were pooled, then the score for a genomic feature used to sort the sites into true (experimental) and false (control) integration sites. The ROC curve plots the true positive rate on the vertical axis versus the false positive rate on the horizontal axis. Conceptually, the curve can be constructed by beginning with a cutpoint (the value of a predictor—for example, gene density) that is higher than the highest value for any site. The ROC curve is started at (0,0). The cutpoint (gene-density value) is then moved down in stages. The curve is extended from lower left to upper right, taking a step in the vertical direction for each correct call (integration site), and taking a step in the horizontal direction for each false call (random control; Figure 1A).

**Figure 2:**
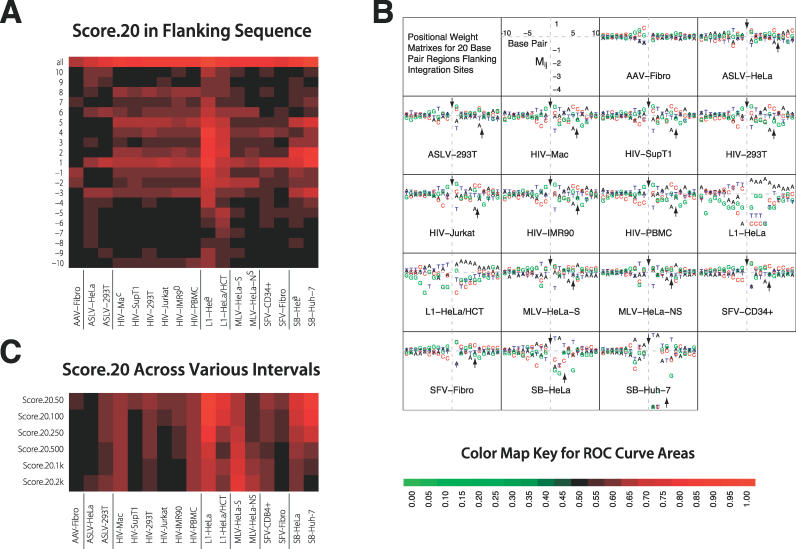
ROC Areas Describing the Effects of DNA Sequences at the 20 bp Surrounding the Point of Integration (Named “score.20”) (A) Heat map of ROC areas describing the influence of sequences at the point of integration. The key at the bottom indicates the color code for ROC values in this and subsequent figures. The top row indicates the summed effect over all the bases in the score.20 motif, and the individual bars below show the area for each individual base in the motif. The site of integration in each case was between base −1 and 1. (B) PWMs for sequences at the point of integration. Bases shown above the line were favorable for integration; those below were unfavorable. The integrating elements differ in the symmetry of the score.20 PWM at the site of integration. The points of joining on the top and bottom DNA strands have been determined experimentally for some cases, and where available are shown by the arrows in Figure 2B. For most of the elements, the sequences are approximately 2-fold rotationally symmetric through an axis between the points of joining on the two strands—this is because, in these cases at the two ends of the element DNA, the DNA breaking and joining steps mediating integration are identical. The exceptions are L1 and AAV, for which the points in the target DNA for joining of the two ends of the element do not have a consistent relationship. Note that the values given for M_ij_ are the logarithms of the relative frequency of nucleotide i at position j among the integration sites compared with its value among the random controls. Thus, if nucleotide i almost always appears in position j in integration sites, M_ij_ will approximate log(2) − log(1/4) ≈ 1.4, while if it appears only once in 128 integration sites M_ij_ will approximate log(1/128) − log(1/4) ≈ −3.5. (C) ROC values for score.20 considered over longer genomic intervals. The number after score.20 on the vertical axis indicates the length in bp, then in kb (the later indicated by k).

Thus the area under the curve is 1.0 when all integration events have higher values for the feature than any control event, and 0.0 for the opposite case. When the area is 0.5, it is equally likely that either has a higher value—thus ROC values near 0.5 are consistent with having no predictive value. As is described in detail in the Text S2, in a few cases additional techniques were used to analyze some parts of the data. A major advantage of the ROC approach is that the effects of different variables can all be scored using a single measure, and then potential interactions or redundancy among variables can be evaluated.

Since there are 17 datasets and several hundred descriptions of genomic features in the analysis, a compact representation of these associations is needed. An overview is provided by the boxplots of the improvement over chance performance as measured by the area under the ROC curves in Figure 1B. This improvement is presented as the absolute value of the difference between the area under the ROC curve and 0.50. Values around 0.0 indicate no useful predictive information for this feature; values near 0.5 indicate that the feature is nearly perfect in separating integration sites from random controls.

As can be seen by comparison of the means (Figure 1B, heavy bars), most of the genomic features exerted a detectable influence on integration targeting. The biases detected here and discussed below were generally highly statistically significant. Exceptions are noted in the text. The scatter, as shown by the whiskers and individually plotted extreme points, emphasizes the differences among datasets. Somewhat unexpectedly, the score.20 ROC value, which reports the effects of the sequence at the 20 bp surrounding the point of integration, shows the strongest effect of any variable.

The influences of each of these variables on integration are considered individually in the next several sections, then combined effects are assessed.

### Effects of Nucleotide Sequence at the 20 bp Surrounding the Point of Integration (score.20)

**Figure 3:**
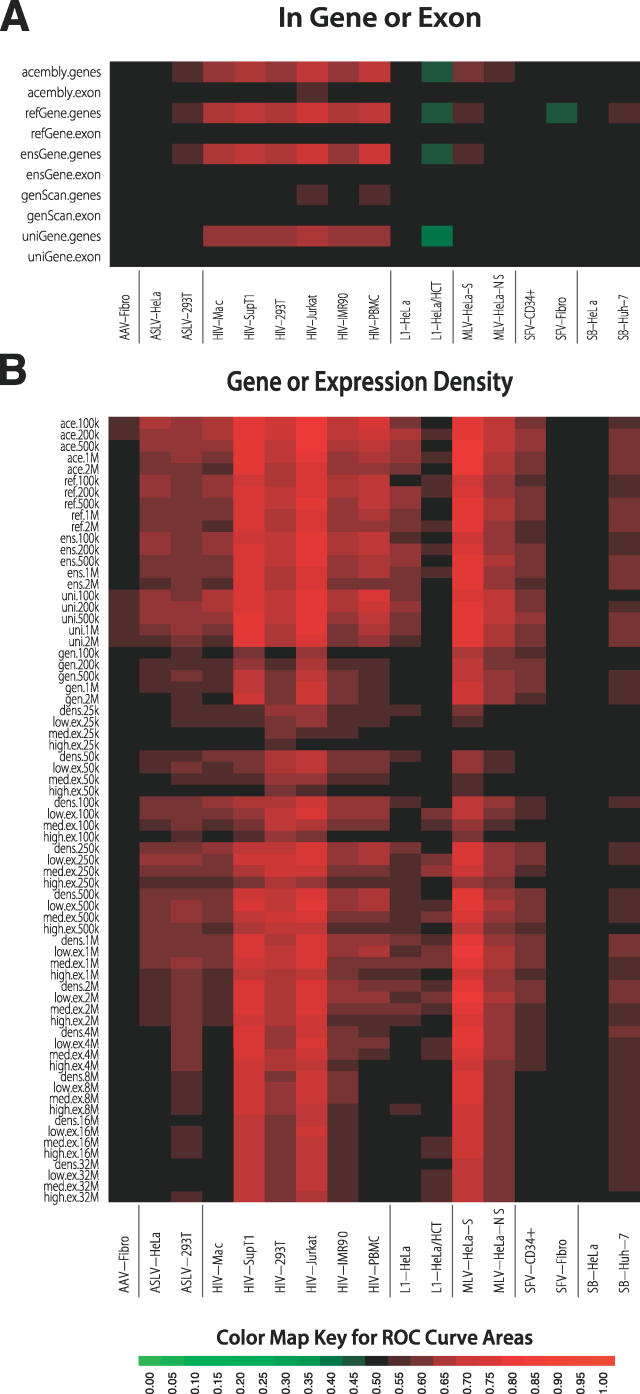
ROC Areas Describing the Effects of Gene-Associated Features on Integration Frequency (A) ROC areas describing the effects of integration within a gene or exon. The databases studied were as indicated. The geneScan database is solely computational, possibly explaining the divergence of ROC areas from the other gene calls. (B) ROC areas describing the effects of gene density or expression density on integration frequency. To calculate the expression density, each gene in an interval was assigned three scores of zero or one according to whether it was 1) in the upper half, 2) in the upper quarter, or 3) in the upper 12.5% of all genes scored in a transcriptional profiling analysis. Transcriptional profiling was carried out using Affymetrix arrays for each of the cell types studied (accession numbers for array data are in Table S1). For each of the three cutoffs, the expression scores for all the genes in each genomic interval were then added together and divided by the interval width to generate the expression-density measure counting the number of expressed genes for that interval.

**Figure 4:**
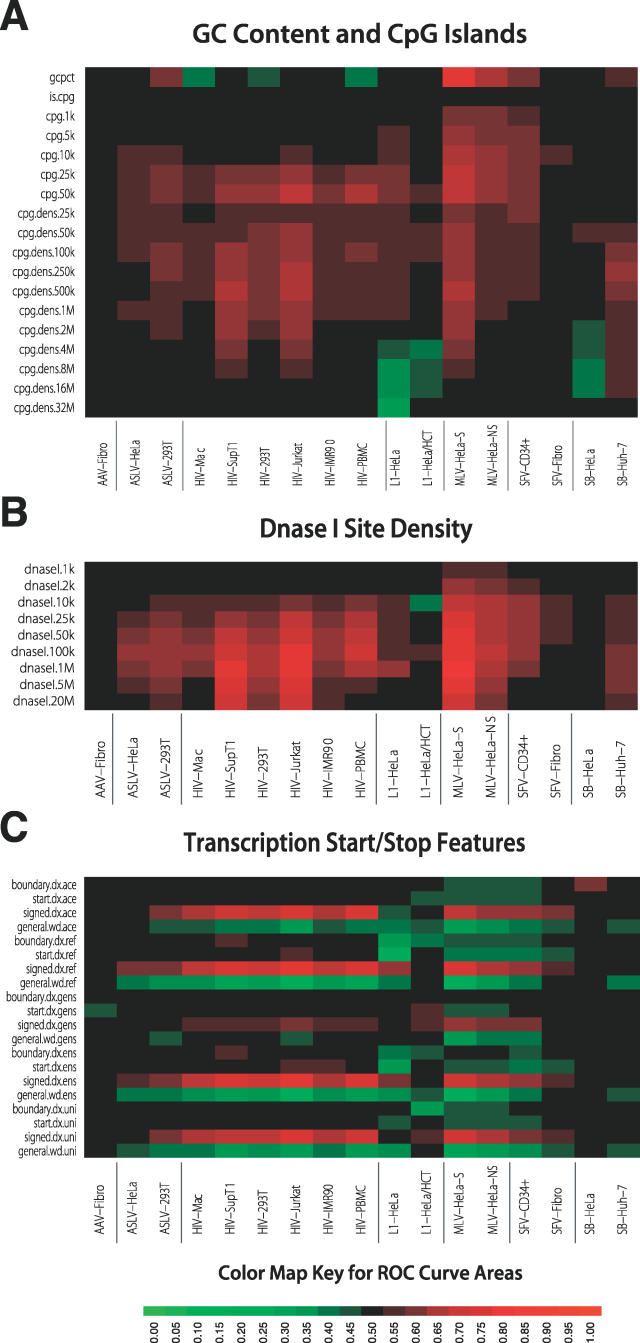
ROC Areas Describing the Effects of Genomic Features on Integration Frequency (A) ROC areas describing the effects of G/C content and CpG islands on integration frequency. CpG islands are on average 764 bp in length. (B) ROC areas describing the relationship between DNase I site density and integration frequency over intervals of different sizes. Each DNase I cleavage site is measured as a single point of cleavage on the human genome. (C) ROC areas describing the effects of proximity to gene boundaries on integration.

Figure 2A shows the ROC areas based on the score.20 sequences illustrated as a heat map. In this and later displays, red indicates favored integration for the feature tested and green disfavored integration. Intensity of color indicates the magnitude. Figure 2B shows the favored sequences plotted to illustrate the weights on the score.20 positional weight matrix (PWM), which describes the log ratio of the frequency of each of the four bases at each position to the frequency in matched random controls. The standard errors for the ROC curve areas in Figure 2A are all smaller than 0.05 (and most are smaller than 0.015); perceptible differences are usually highly statistically significant. As an example, the *p*-values for the score.20 ROC curve areas (Figure 2A, top row) are all less than 10^−14^, and this holds for ROC curves in Figures 3–4 as well.

Datasets for each type of integrating element show closely similar favored bases, despite the differences in cell types and experimental conditions. This fits with the expectation that the score.20 PWMs mostly represent favored sites of binding or catalysis for the element-encoded recombination enzymes, as suggested in previous work. For L1, the endonuclease that initially cleaves the target site DNA strongly favors 5′-AAATTT-3′ sites [35], which explains the strong correlation between integration and the L1 score.20 PWM [21,22]. For the SB DNA transposon, integration is strongly favored at 5′-TA′3′ base pairs [23,24], and this is similarly reflected in the activities of purified transposases related to SB [37,38]. Weaker but still quite significant effects are seen for the retroviruses [31–34]. AAV integration shows the weakest effects of score.20, potentially a consequence of integration at spontaneous DNA double strand breaks [28].

Many of the questions surrounding integration frequency involve genomic segments such as promoter regions, where one is interested in knowing how likely integration is in such a region, and how important different features are in directing integration towards that region. Such questions can be answered directly by calculating the expected number of integration events at each bp in a region and adding them together to obtain the expected number of integration events for the region. For some annotations, this can be computationally burdensome. However, a less computationally burdensome approximation can be used when integration events are sparse in the genome. To determine whether the score.20 PWM helps identify longer intervals that host integration events, intervals from 50 bp to 2 kb containing integration sites or controls were scored. Each base in the interval was treated as the edge of an integration site; then all such windows were scored over the interval, and the interval scores summed. The summed values were then tested for their ability to sort experimental integration sites from controls and the results were presented as areas under ROC curves (Figure 2C). Thus, this procedure tests whether favored primary sites are clustered in the genome.

Comparison of the top row of Figure 2A (ROC areas describing the effects of the full score.20 motif) to the rows in Figure 2C shows that the score.20 PWM distinguished integration sites from controls much less efficiently when larger intervals were tested for all of the integrating elements. This may seem an obvious result given the specific motifs favored for integration (Figure 2B), in which a single base shift can change a highly favorable motif to an unfavorable one. However, if particular regions are rich with favorable motifs, the average score over an interval may well predict integration. For eleven of the seventeen datasets, a graded reduction in ROC area is seen with increasing length of the interval considered. This is as expected if high-scoring matches to the score.20 PWMs are relatively common, so that substantial numbers of high scores are encountered almost as often in a randomly selected interval as in an interval containing an integration site. Surprisingly, the MLV values increase with longer interval sizes, though not back up to the original score.20 values in Figure 2A, indicating some degree of clustering of favored motifs. Thus, the influence of score.20 is mostly reduced at longer lengths scales, though even with 2-kb intervals for many of the integrating elements the effect was still discernable.

### Integration in Transcription Units and the Effect of Gene Activity

Figure 3A shows a heat map for the area under the ROC curves, summarizing the increase in integration frequency within TUs and exons. Several human gene catalogs are available, so we repeated the analysis for five of them. The “exon” ROC areas showed no discernable effect and will not be considered further. However, ROC areas for TUs showed strong effects that differed among the datasets. All the HIV datasets showed favored integration in most of the TU calls, which is consistent with previous reports [8–16]. Of the other retroviruses, the two MLV datasets and ASLV-293T showed weak favoring for several of the TU calls [9,16], indicating weak association, while SFV showed no association, or in one case negative association [18]. For SB, one dataset showed a weak association with refGenes, but all other measures were negative. In previous literature there was disagreement on whether L1 favored integration in TUs [21,22]. By the ROC approach used here, TUs were either unfavorable or had no influence. Similarly AAV integration targeting was not affected by TUs.

Figure 3B shows a similar heat map based on ROC areas, this time summarizing the effects of gene density in genomic intervals of different sizes and a related measure that adds transcriptional activity to yield “expression density.” As before, the five sets of gene calls are compared. The effects of each feature were tested over intervals from 100 kb to 4 Mb. All of the datasets were at least weakly positive for at least a few of the measures. Particularly strong effects were seen for the HIV datasets in lymphoid cells or cell lines, and for the MLV dataset that was selected for expression after infection (MLV–Hela-S). Also favored, though less strongly, were the HIV datasets in other cell types and the remaining MLV dataset. Of the HIV datasets, the one showing the weakest response was from nondividing macrophages—together with other measures, this is consistent with a model in which the nondividing state of these cells diminished integration in active TUs [14]. There was no clear pattern of interval size, type of gene call, or expression level. This suggests that features broadly associated with high gene density were most significant.

The two ASLV datasets showed weak favoring of gene-dense and intensely expressed regions. AAV, SFV, and SB showed the weakest responses—for AAV, it was unclear that there was any significant favoring of integration near or within these features.

Note that each integration site dataset was analyzed versus transcriptional profiling data for the cell type hosting the integration events (Table S1). This was important, because previous work has shown that, for HIV, tissue-specific transcription is associated with tissue-specific patterns of integration, though the strength of the bias is modest [10].

### G/C Content and CpG Islands

We next investigated the effects of G/C content and proximity to CpG islands (Figure 4A). Regions of high G/C on average are gene-rich and have short introns, high frequencies of Alu repeats, low frequencies of LINEs, high frequencies of CpG islands, and replicate early. Regions of low G/C content are typically opposite in these features [1]. CpG islands are defined by clusters of the rare dinucleotide CpG that are undermethylated and are commonly associated with gene regulatory regions. The top row in Figure 4A shows the ROC areas describing the response to G/C content for the seven integrating elements. The two MLV datasets show a strong favoring of regions of high G/C for integration. In contrast, three of the HIV datasets show a favoring of low G/C, which is paradoxical—HIV favors integration in gene-rich regions, which are typically rich in G/C, but instead A/T is favored. As is discussed below, this may reflect the action of the cellular HIV integrase-binding protein PSIP1/LEDGF/p75 [12]. The other datasets showed weaker and less consistent responses to G/C content.

The remaining rows indicate the response to CpG island density over increasing length genomic intervals (from 1kb to 32 Mb). For short intervals, proximity to CpG islands correlates with proximity to regulatory regions, while for intervals long enough to span many genes, the CpG island density correlates with gene density (e.g., [40]). Inspection of the ROC areas for short intervals (1–10 kb) shows that integration is enriched near CpG islands most notably for MLV, which is consistent with favored integration near regulatory regions as reported previously [9,10,16]. One of the two SVF datasets and one of the two L1 datasets show weaker but detectable enrichment. The other elements were not responsive to nearby CpG islands, either favorably or unfavorably. For the longer genomic intervals, HIV and MLV showed the highest ROC areas, as expected from their known preferences for TUs (HIV) and gene 5′ ends (MLV). ASLV showed weaker positive ROC areas. AAV and SB showed no consistent favoring of CpG islands at long segment lengths, while L1 showed negative correlations. Thus integration scores for CpG islands analyzed over long intervals paralleled the responses to gene density and transcriptional intensity.

### DNase I Cleavage Sites

The response of the seven integrating elements to mapped points of DNase I cleavage are summarized in Figure 4B. DNase I hypersensitive sites in chromatin have previously been associated with transcription factor binding sites, CpG islands, and gene control regions [41]. Older literature from the retroviral field suggested an association of MLV integration with DNase I hypersensitive sites [42]. However, a more recent study suggested that MLV, and not HIV or L1, integration was more frequent in 2-kb intervals enriched for DNase I cleavage sties [16]. ASLV showed a weak but positive correlation [16]. This indicated that association with DNase I sites is mostly an MLV-specific feature at this length scale, paralleling the preference of MLV for integration near gene 5′ ends and CpG islands.

Figure 4B presents a study of all seven integrating elements analyzed over intervals ranging from 1 kb to 20 Mb. For short segment lengths (1–2 kb), only the MLV datasets showed ROC areas indicating favored integration near DNase I cleavage sites. As the segment lengths become longer, the density of DNase I cleavage sites increasingly parallel the density of TUs and gene regulatory regions. Thus, both HIV and MLV are strongly positive when analyzed over longer intervals, and most of the other datasets are weakly so. AAV and one of the SB datasets show the lowest ROC values relative to this measure.

### Integration Near Transcription Factor Binding Motifs

The effects of proximity to transcription factor binding sites on integration are summarized in Text S2 (p. 18). This is of interest since it is possible that direct binding of integration complexes to transcription factors might promote integration via a tethering interaction [7,12]. We analyzed the TRANSFAC database, which contains 546 PWMs describing DNA binding sites for transcription factors. To assess effects of each on integration, the 2-kb interval centered on each integration site or random control was given a score based on the best single match to the PWM, and this score was used to generate an ROC area describing effects of that PWM. Many PWMs showed detectable positive or negative associations with integration. The most notable was for the two MLV datasets, where a substantial fraction of all PWMs showed positive association. As is discussed below, the TRANSFAC PWM results did not have strong predictive value when analyzed together with other genomic features such as gene density and proximity to gene boundaries. However, future studies using more sophisticated scoring functions may yet reveal informative associations between TRANSFAC PWMs and integration frequency.

### Proximity to Transcription Start and Stop Features

Several measures were used to compare integration frequency for the experimental and matched random control sites near transcription start and stop features as ROC areas (Figure 4C). The measure “boundary.dx” measures the distance to the nearest gene 5′ or 3′ end. The green coloring seen for several datasets indicates an ROC curve area of less than 0.50, which is the result of integration sites tending to have shorter distances to the nearest gene 5′ or 3′ end than a matched random control site. However, most of the cells are black or nearly so, which reflects ROC curve areas near 0.50 and implies that there is little correlation with integration. “Start.dx” indicates the distance to the nearest gene start sites. Again, integration sites tend to be closer to start sites than their matched random controls, and so is sometimes shown as more intense green. “Signed.dx” scores sites by a function reflecting higher probability of integration near start sites, so increased integration near start sites results in a positive correlation and more intensely red coloring, as is seen for several datasets. “General.width” is a measure of the length of the interval, defined by the nearest transcriptional start and stop features, that also contains the integration site. Large values thus reflect gene-sparse regions, and are inversely correlated with gene density. Each of the measures was tested over the five collections of human gene calls.

For the two MLV datasets, measures reflecting proximity to gene 5′ ends (start.dx, boundary.dx, and signed.dx) showed significant ROC values, as expected from previous work. The analysis presented here establishes that these results were mostly independent of the gene calls used. For HIV, start.dx and boundary.dx showed little predictive value, consistent with gene 5′ ends not being particularly favorable for HIV integration. The signed.dx value reflects integration in the 5′ regions of TUs, and so is positive for the HIV datasets. Similarly, the general.width measure, which is inversely related to gene density, was negatively correlated with HIV integration. ASLV and SFV showed weak responses to signed.dx and general.wd, reflecting favoring of integration in gene-rich regions, but no consistent favoring of gene 5′ ends. However, SFV did show some favoring of gene 5′ ends for the CD34+ dataset but not for the fibroblast dataset, indicating a possible cell type–specific difference. L1 showed few consistent responses, though several of the general.dx and boundary.dx calls had predictive value, reflecting potential weak favoring of integration in gene-dense regions. AAV and SB integration showed no consistent responses to any of these measures, indicating that gene boundaries do not strongly affect integration in these datasets.

### Improved Models Incorporating Score.20 Together with Other Genomic Features

**Figure 5:**
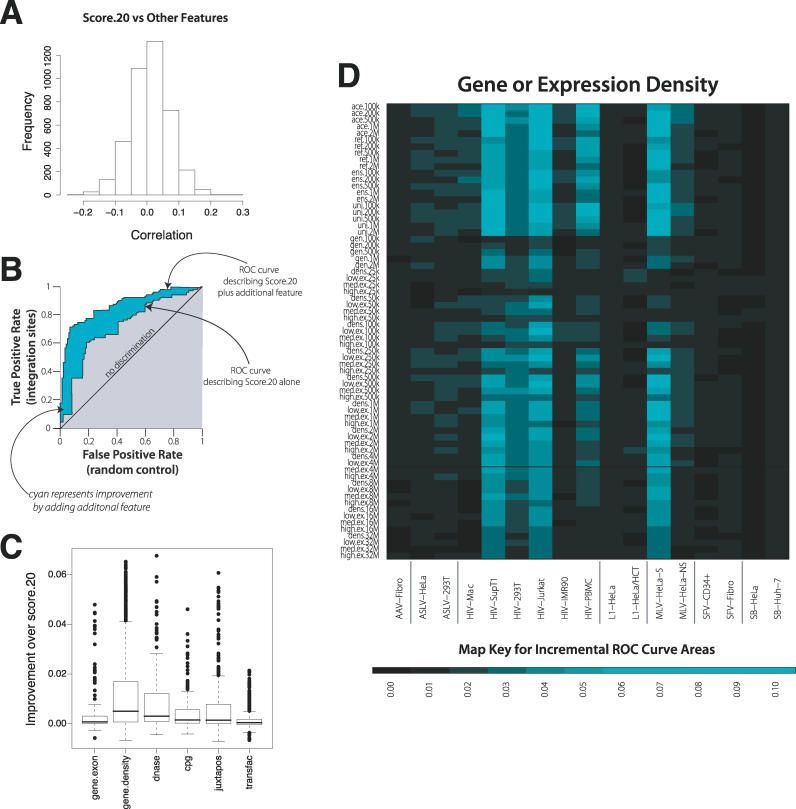
Improved Prediction due to Adding Additional Genomic Features to the score.20 ROC Values (A) Lack of correlation between score.20 and other measures. (B) Diagram of the analytical method, illustrating the improvement of an ROC score by addition of a second predictor to the score.20 value. (C) Box plots describing the improvements in ROC area resulting from combining other genomic features with the score.20 measure. The ROC area increments are modest, because the ROC curve based on the combination of score.20 and another feature can only vary between the value for the score.20 prediction and 1.0. (D) Heat map of increases in ROC areas resulting from adding the gene density measures to the score.20 values for each integrating element. The color code for improvements in ROC areas is indicated at the bottom.

We next investigated how combinations of genomic features affect integration. As discussed above, the score.20 PWM was most effective for distinguishing authentic integration sites from the random controls (Figure 2). Thus we began by asking whether the other genomic features were merely redundant with score.20 by analyzing the correlation of the other features with score.20 (Figure 5A). Little correlation was detected, suggesting that a predictor of integration targeting constructed based on score.20 together with other features could substantially improve prediction based on either alone.

As a first step to assess the effects of combining features, we used a regression method that fit both the score.20 data and a second genomic feature (see Text S2, pp. 24–52). The fitted value for the integration intensity was then used to calculate the area under the ROC curve describing the joint prediction, and this was subtracted from the curve based on score.20 alone (Figure 5B). Note that the fitting process leads to fitted values that tend to rank the integration sites more highly than the matched random controls, so the ROC curve areas based on these fitted values are all greater than 0.50. The difference between the two curves thus describes the improvement in prediction due to inclusion of the additional genomic feature. The standard errors for the ROC curve area differences in Figure 5C and 5D are all smaller than 0.02 (and most are smaller than 0.01); perceptible differences are usually highly statistically significant.

A box plot summary of the improvements in ROC areas is shown in Figure 5C. Evidently many features can improve prediction for at least some of the datasets, with measures of gene density and expression intensity showing the greatest effects. To obtain a more detailed view, these “improvement” values can themselves be plotted as heat maps. The incremental improvements over score.20 by inclusion of gene-density measures are shown in Figure 5D. This map generally resembles the original heat map of ROC areas for gene density without consideration of the score.20 contribution, reinforcing the idea that the two are independent predictors of integration frequency. A complete set of heat maps, which allows the incremental contribution of each genomic feature to be assessed, is included in the Text S2.

### Comprehensive Models Incorporating All Types of Genomic Features and Their Combined Effects

We next sought to combine all the genomic features together into a single model. Regression methods can be used to fit multiple features at once, but given the number of features and datasets to be explored here there are more than 10^70^ possible combinations of variables to form models. For this reason, we investigated combined effects using an approach based on Bayes model averaging (BMA) [43]. Models with high posterior probability were collected and used to evaluate the importance of the various features; the posterior mean of the regression coefficient for a genomic feature summarizes the effect of that feature when in combination with other features in the dataset. More detailed methods can be found in Text S2 (pp. 24–52).

The contributions of each class of features to each of the integration models are summarized in Text S2 (p. 40). Consideration of the genomic features in the context of the full BMA model reinforces that the score.20 indicator and the other types of genomic features make independent contributions. However, now several of the genomic features show lower relative contributions (e.g., TRANSFAC PWM scores and juxtaposition with transcription start and stop features), suggesting that these are largely redundant with other measures. Heat maps of the effects of genomic features as reported by the BMA model are shown in Text S2, pp. 42–50. Further modeling using the machine learning program RandomForest can be found in Text S2, pp. 51–53, which yielded a generally similar picture. We return to selected combination effects below.

**Figure 6:**
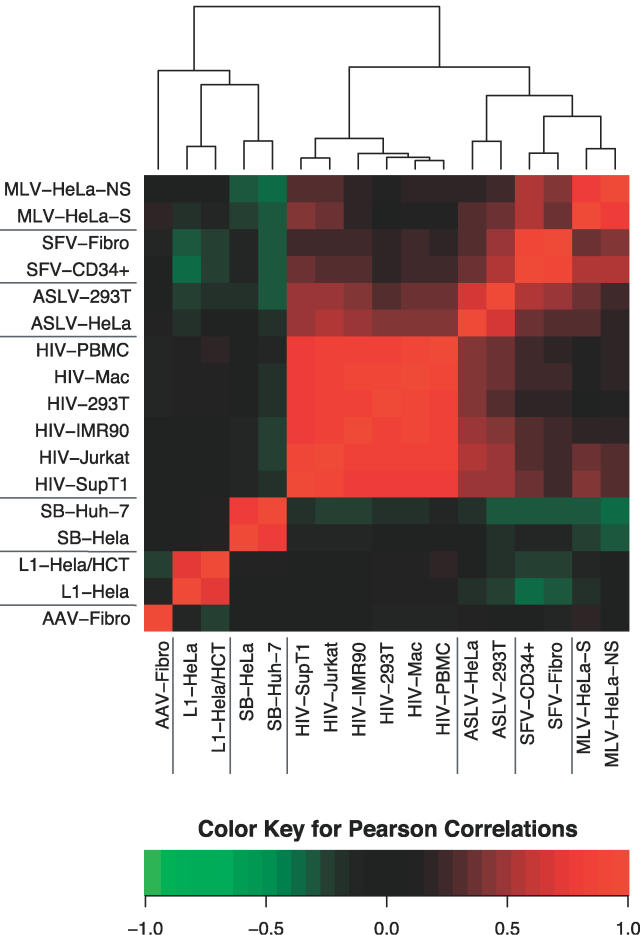
Clustering the Comprehensive BMA Integration Site Models Red indicates positive correlation and green indicates negative, as illustrated by the key at the bottom of the figure. The models were clustered in both the *x*- and *y*- directions, so the graph is symmetrical along a line from lower left to upper right. See Text S2 for more details.

We then used the full BMA model to specify the relationships among the models for the different integrating elements (Figure 6). To generate values to allow comparison, a sample of random genomic sites was scored for the logarithm of the odds of integration using each of the BMA models. The correlations among scores are displayed in false color in Figure 6. Green corresponds to negative correlations and red corresponds to positive. The results were subjected to hierarchical clustering to highlight the similarities among datasets. Inspection of the branching pattern shows that the first major division is between the retroviruses and other groups. Within each of these branches the different element types were well-resolved, with slight resemblance among the retroviruses, but little between the retroviruses and SB, L1, and AAV. Thus the BMA models (Figure 6) grouped the 17 datasets tightly by element type, supporting the conclusion that integration site selection is dominated by the element-encoded recombination enzymes that carry out the integration reaction. Factors such as cell type, selection for expression after integration, and cell division have detectable but much weaker effects.

## Discussion

The genomic features that had the strongest effects on each type of integrating elements are summarized below, with an emphasis on new findings in this study. Subsequently, general conclusions and uses of the quantitative model are considered.

### L1

For the non-LTR retrotransposon L1, the score.20 PWM allowed near-perfect sorting of integration sites from control sites. When longer genomic intervals were considered, the effect of score.20 was still detectable, though diminished. Addition of other genomic features to a model based on score.20 alone using BMA showed little or no further improvement in ROC areas. Thus L1 integration site selection is dominated by the sequence at the point of integration, and otherwise is mostly nonspecific. Previous studies had noted the strong conservation of the nucleotide sequence at L1 integration sites [21,22]; this study establishes that the local sequence had strong effects on integration genome-wide.

### Sleeping Beauty

For SB as well, the score.20 PWM was the predominant determinant, though the effect was reduced when longer intervals were used for comparison. The dropoff with interval size was steeper than for L1, in part because the main determinant of the favored site is relatively short (the dinucleotide 5′-TA-3′). Addition of other genomic features to a model based on score.20 alone resulted in only slight improvements. Unexpectedly, the genomic features important beyond score.20 identified in the BMA model diverged for the two SB datasets. Proximity to CpG islands, particularly analyzed over relatively long genomic intervals, was anticorrelated with integration in the Hela-cell dataset, whereas it was weakly positive in the Huh-7 dataset. Gene density was positively correlated with integration in the Huh-7 dataset only. This suggests possible cell-type–specific differences in SB integration. However, more data would be helpful to strengthen this idea, because the two SB datasets came from different laboratories and integration sites were cloned using different methods [23,24].

### HIV

Previous studies of HIV integration revealed that active TUs were favored integration targets, and that trend was recapitulated by a variety of measures in this data. Previously, proximity to DNase I cleavage sites was reported not to be associated with HIV integration over short (2-kb) genomic intervals [16]. Analysis presented here shows that DNase I sites correlate positively over longer intervals, probably because of the correlation of both HIV integration sites and DNase I cleavage sites with gene-dense regions. Other measures, such as CpG islands and transcription start/stop features, also correlate positively with HIV integration at long interval sizes for the same reason. A substantial effect of score.20 could be detected in the initial phase of the analysis based on scoring individual base pairs, but this was considerably diminished, and for three datasets eliminated, when effects of score.20 were considered over longer genomic intervals.

The BMA model unexpectedly revealed a strong correlation between HIV integration and A/T-rich sequences (Text S2, p. 47). This is opposite to simple predictions based on favoring of integration in gene-rich regions, because gene-rich regions are G/C-rich. However, gene density is accounted for in the BMA models, so the effect of base composition is in addition to gene-density effects. The cellular PSIP1/LEDGF/p75 protein, which binds tightly to HIV IN, has an A/T hook DNA binding motif, which would be expected to cause accumulation of PSIP1/LEDGF/p75 on A/T-rich DNA. Depletion of cells for PSIP1/LEDGF/p75 results in increased HIV integration in G/C rich regions [12]. The finding of high A/T density at HIV integration sites (when controlling for other effects) across all six HIV datasets suggests that PSIP1/LEDGF/p75 likely influences HIV integration in all of the cell types tested.

### MLV

MLV integration showed an association with gene 5′ ends, CpG islands, and DNase I hypersensitive sites in previous studies where short window sizes were used for comparison [9,16]. Analysis presented here shows that this effect often becomes even more pronounced when larger window sizes are analyzed (25 kb to 2 Mb). The comparisons over longer intervals likely capture effects due to both nearby gene 5′ ends and more global gene density. Analysis of the effects of these features in the BMA model showed considerable redundancy with transcription factor binding sites (TRANSFAC PWMs), consistent with a model in which these features are at least partially redundant. Score.20 had a clear-cut effect on MLV integration, and this was reduced when score.20 values were summed over longer intervals, though for unknown reasons the summed effects of score.20 were actually greater with longer intervals sizes, suggesting autocorrelation of favored sites.

The effects of selection were prominent in the comparison of the two MLV datasets, as described previously [16] and analyzed in more detail here. Measures of association with gene density, expression density, DNase I cleavage, and CpG islands were all more pronounced in the selected dataset (MLV–Hela-S). This is consistent with the idea that integration near these features results in more efficient proviral gene expression, so that after selection for gene expression, proviruses near these features become enriched in the population. A similar trend has been reported for HIV [15,16].

### ASLV

ASLV integration showed weaker (though still detectable) favoring of genomic features associated with genes and gene density, as suggested previously. For example, in the analysis of ASLV integration near DNase I sites over longer genomic intervals, which is newly reported here, a consistent positive correlation was seen in both ASLV datasets. The score.20 PWM analysis showed significant effects on integration site selection over short intervals, but this was mostly eliminated in analysis over longer genomic intervals.

### SFV

Similarly with SFV, comparatively weak association was seen with genes, gene density, and associated features. Some weak association was seen with DNase I sites and CpG islands over longer genomic intervals. Score.20 scored relatively weakly compared with other elements, and effects of score.20 were reduced or absent in the comparison over long windows.

For SFV, this analysis emphasized the cell-type–specific differences between the two datasets. The association with gene-related features was noticeably greater for sites from CD34+ stem cells than for sites from fibroblasts. In the previous analysis of these datasets, pooled SFV sites from both cell types were reported to be enriched near gene 5′ ends and CpG islands. The analysis here discloses that this is almost entirely due to the contribution of the sites from the CD34+ cells, while those from fibroblasts showed no such bias. Similarly, with proximity to DNase I cleavage sites, analyzed here for SFV for the first time, there is a positive correlation but the effect is much stronger in CD34+ cells.

### AAV

AAV vectors are unique among the integrating elements studied here because AAV DNA is believed to be integrated by host DNA repair enzymes acting at spontaneous DNA double-strand breaks. The AAV score.20 PWM showed enrichment for G/C at positions −1 to −3, and this was the most prominent bias detected. Possible weak favoring of integration near gene-rich regions was also seen. An intriguing possibility is that these biases reflect a nearly random distribution of spontaneous chromosomal double-strand breaks. However, it is also possible that these biases reflect a greater likelihood of these sites participating in the repair reactions mediating AAV integration. Of all the datasets studied, the AAV vector data showed the least favored integration in TUs or gene 5′ ends. Potentially this increases the attractiveness of AAV as a gene therapy vector. However, a study of AAV integration in mouse liver [27] suggested a strong association with gene 5′ ends and CpG islands, quite different from the dataset studied here. Thus, further data on AAV integration in different cell types would be useful.

### General Conclusions and Uses of the Models

As discussed above, the analysis particularly emphasized the importance of the sizes of genomic segments used for comparing genomic features and integration intensity. At 3.5 billion bp, the human genome is so large that the effects of different genomic features on integration may change or even be opposite depending on the length scale in question. This was evident in several ways. Changing the size of intervals used to collect values for genomic features typically changed the ROC scores. For example, the effects of CpG island density on L1 elements (Figure 4A) scored as weakly positive over shorter genomic segments (25 kb to 1 Mb), but was negatively correlated over long intervals (4 Mb to 32 Mb). Conversely, for several retroviruses, some effects of gene density, expression intensity, DNase I site density, and CpG island density became more significant with increasing interval length. In another example, summing integration scores for each base across longer intervals also resulted in different ROC values. Figure 2C shows that the score.20 index had much less influence on ROC scores when 2-kb regions were compared instead of 20-bp segments. Thus, to answer the question “What genomic features influence integration of new DNA?”, the length scale of interest must be carefully specified.

Going forward, the ability to predict integration intensity for each base in the human genome will be useful as a tool for detecting new influences on target site selection. For any new genomic feature that is found to influence integration when analyzed in isolation, it is now possible to assess whether the feature contributes information independent of previously studied features. Integration intensity can be predicted by either the standard model described here or by the standard model plus the new feature, and the predictions of the two models compared with experimental integration data. Improved prediction by addition of a new feature establishes the importance of that feature. Conversely, lack of improvement indicates that the new feature is redundant with previously known features. This method should be quite useful in evaluating the influence of newly annotated genomic features on integration. For example, a large number of new types of annotation are now available for the 1% of the human genome in the ENCODE regions [44], and it will be interesting to use the integration models to evaluate their effects.

## Materials and Methods

### Data Analysis Strategy

The data analysis is based on a “nested case control” strategy (for a review, see [45]) that uses a collection of integration sites (in the role of “cases”) along with control sites (the “nested controls”) sampled from the genome (the “cohort”) to make inferences about the probability of integration at a particular location based on the genomic features that characterize that location. This strategy depends on the relationship of a log-linear model of location-specific counts of integration to logistic or conditional logit models that discriminate between actual integration events and control sites, viz. the same parameters that govern the effect of a genomic feature on integration govern discrimination between actual and control sites. The logistic model is appropriate for random genomic controls, while the conditional logit model is appropriate when a set of controls is matched to each integration site (the matching of which is done to control for possible biases in the recovery of integration events). A more detailed description of the statistical basis for this analysis can be found in Texts S1–S3.

### Software Used

The data were analyzed using the R language and environment for statistical computing and graphics version 2.3.0 (R Development Core Team, 2006) and several contributed packages. Bayes model averaging used the package BMA, and Random Forest computations used the randomForest package. Parallel processing was implemented using the snow package.

### ROC Curve Areas

Empirical ROC curve areas [46] were calculated for datasets that used random genomic controls. When matched controls were used, each integration site was compared only with its matched controls to determine the proportions of controls whose values equaled or exceeded that of the integration site.

### Annotation of Genomic Features

Integration site datasets were obtained from the US National Center for Biotechnology Information. For the dataset of [17], information on the location of the integration site relative to the deposited genomic sequences was obtained from the authors. Locations of genes and exons and G/C percent were based on the May 2004 tables (hg17) from the Annotation database of the GoldenPath Web site (http://hgdownload.cse.ucsc.edu/goldenPath/hg17/database/). Computations of gene density, and of juxtaposition of transcription start/stop features, were based on those same tables. The computation of DNase I site density was based on a table of DNase I sites obtained from [16]. The expression density measurements used transcriptional profiling data matched to each cell type. Accession numbers for these datasets are specified in Table S1.

### Data Sharing

Software and processed data are available upon request.

## Supporting Information

Table S1 Sources of Gene Expression Data Used in the Analysis (55 KB XLS)

Text S1 ROC Curve Construction Explained (230 KB PDF)

Text S2 Screening Effects on Retroviral Integration (332 KB PDF)

Text S3 Clustering Transcription Factor PWMs (43 KB DOC)

## Abbreviations

AAV: adeno-associated virus
ASLV: avian sarcoma–leukosis virus
BMA: Bayes model averaging
LINEs: long interspersed nuclear elements
MLV: murine leukemia virus
PWM: positional weight matrix
ROC: receiver operator characteristic
SB: Sleeping Beauty
SFV: simian foamy virus
TU: transcriptional unit
