# Supplementary material for: Selection of Target Sites for Mobile DNA Integration in the Human Genome
Source: PLoS Comput Biol. 2006 Nov 24;2(11):e157. doi: 10.1371/journal.pcbi.0020157 (PMC1664696; doi:10.1371/journal.pcbi.0020157)
Supplement: Text S1 — (230 KB PDF) [file pcbi.0020157.sd001.pdf]

ROC Curve Construction Explained

The following diagrams show the steps needed to construct an ROC curve for discriminating integration sites from genomic control sites using a genomic feature. Here the feature considered is the loglikelihood score derived from position weight matrices (PWMs) for the 20 base pairs flanking an integration (or control) site (aka `score.20`). The data used are for the SB-Hela integration site.

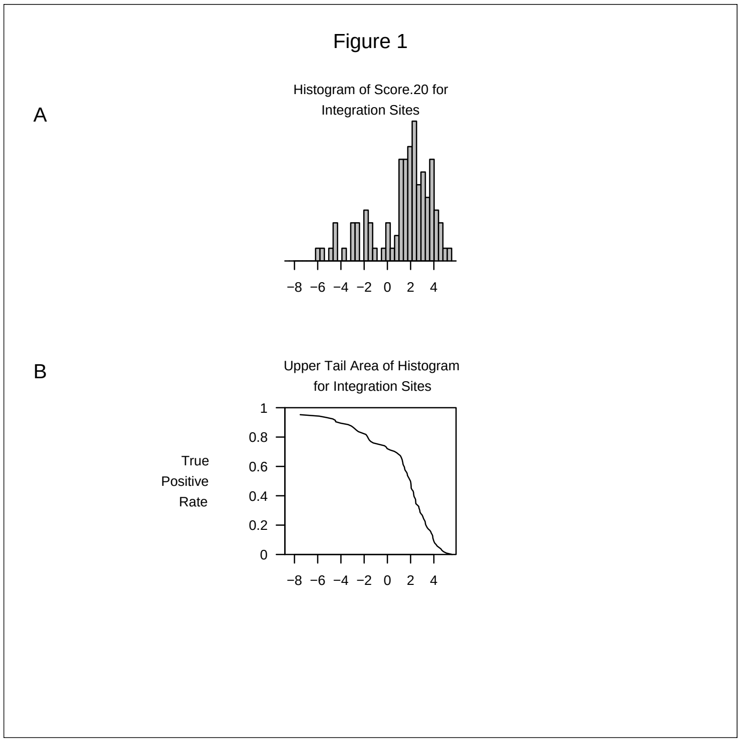

First, the `score.20` values for the integration sites are tallied to create the histogram (**Figure 1A**) and the upper tail areas of the histogram (**Figure 1B**) , which shows the fraction of integration sites (vertical axis) that have values for the feature that exceed a given value (horizontal axis). This fraction is sometimes called the *true positive rate*. To obtain the ROC curve, the *false positive rate* must also be obtained in a similar manner using data from the randomly sampled genomic sites.

The ordinary histogram for randomly sampled genomic sites is shown in panel **Figure 2E**, but is rotated 90° clockwise. Likewise, the plot of upper tail areas of the histogram is created for randomly sampled genomic control sites shown in panel **Figure 2F** is rotated. (Both panels of Figure 1 are repeated in Figure 2 as panels **C** and **A**.)

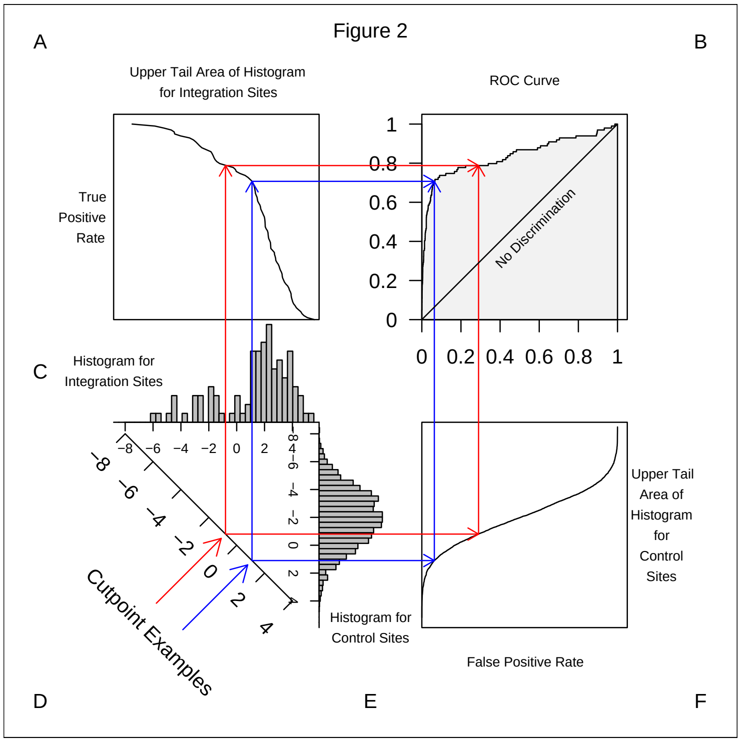

The ROC curve is constructed from the collection of true and false positive rates as follows: For every possible cutpoint, the upper tail area or *True Positive Rate* is read from panel **Figure 2A** (call it y), and the *False Positive Rate* is read from panel **Figure 2F** for the control sites (call it x). The (x, y) pair is plotted (**Figure 2B**). This is illustrated for two cutpoints shown by color coded arrows in panel **D**. Following the color coded lines for each cutpoint leads to panels **A** and **F** where the true and false positive rates are read, and then to panel **B** where the pair is plotted. The ROC curve is formed by joining each pair of adjacent (x, y) points with a line.

Notice that if $x = y$ for every pair, then the *line of identity* (labelled *no discrimination* in panel **B**) is obtained. The area under that line is 0.5. The absolute area between the ROC curve and the line of identity is sometimes called the *discrimination*.
