# Supplementary material for: Selection of Target Sites for Mobile DNA Integration in the Human Genome
Source: PLoS Comput Biol. 2006 Nov 24;2(11):e157. doi: 10.1371/journal.pcbi.0020157 (PMC1664696; doi:10.1371/journal.pcbi.0020157)
Supplement: Text S2 — (332 KB PDF) [file pcbi.0020157.sd002.pdf]

Screening Effects on Retroviral Integration

Charles C. Berry

August 14, 2006

Abstract

Seventeen different integration complexes are studied. Genomic features that might predict integration are screened. Among these, many show association with integration targetting in one or more complexes. The strongest effect for each complex is due to the local oligonucleotide sequence as measured by a score based on the position weight matrix for the surrounding 20 base pairs. Predictive models that combine this score with other features are considered, and some substantially improve the prediction of integration targetting beyond that of the twenty base pair score.

Contents

1 DataSets Used **2**

2 Variables Used **3**

3 Measuring Association of Features with Integration **5**

4 Association of Features with Integration Targetting **8**
- 4.1 Gene or Exon 11
- 4.2 Gene or Expression Density 12
- 4.3 Dnase I Site Density 15
- 4.4 GC Content and CpG Islands 16
- 4.5 Transcription Start/Stop Features 17
- 4.6 TRANSFAC scores 18
- 4.7 Positional Weight in Flanking Sequence 21

5 Details of Local Sequence Effects **22**

6 Association of Local Sequence and Other Features **23**

7 Incremental Effects of Features **24**
- 7.1 Gene or Exon 27
- 7.2 Gene or Expression Density 28
- 7.3 Dnase I Site Density 31

7.4 GC Content and CpG Islands 32
7.5 Transcription Start/Stop Features 33
7.6 TRANSFAC scores 34

8 Combined Effects of Features 37
8.1 Regression via Bayes Model Averaging 37
8.1.1 Gene or Exon 43
8.1.2 Gene or Expression Density 44
8.1.3 Dnase I Site Density 46
8.1.4 GC Content and CpG Islands 47
8.1.5 Transcription Start/Stop Features 48
8.1.6 TRANSFAC scores 49
8.2 Fitting via RandomForests ™ 51

References 54

1 DataSets Used

Each dataset used in this analysis has one of two types of control:

match the integration sites were recovered using a restriction enzyme. The control site matches the distance from the nearest restriction site in the direction of transcription.

random The control site is merely a random draw from the genome.

The datasets, the element types of the integrastion cokmplexes (aka *integrants*), their control types, and the number of integration sites studied are listed here:

	Element	Control.Type	Number.of.Sites
AAV-Fibro	AAV	random	434
ASLV-HeLa	ASLV	matched	194
ASLV-293T	ASLV	matched	640
HIV-Mac	HIV	matched	786
HIV-SupT1	HIV	matched	587
HIV-293T	HIV	matched	1185
HIV-Jurkat	HIV	matched	914
HIV-IMR90	HIV	matched	482
HIV-PBMC	HIV	matched	542
L1-Hela	L1	random	92
L1-Hela/HCT	L1	random	127
MLV-HeLa-S	MLV	matched	544
MLV-HeLa-NS	MLV	matched	917
SFV-CD34+	SFV	matched	1751
SFV-Fibro	SFV	matched	962
SB-Hela	Sleeping Beauty	random	99
SB-Huh-7	Sleeping Beauty	random	282

2 Variables Used

The variables used describe genomic features that summarize characteristics of the genomic sequence surrounding the integration (or control) site. For convenience of discussion they are divided into categories.

The categories are:

Genes and Exons Indicator variables for whether the site falls into a gene or an exon. This is abbreviated as `gene.exon` in some displays. There is one for each of several gene annotation schemes labelled as follows:

- **ace** Acembly or AceView `http://www.ncbi.nlm.nih.gov/IEB/Research/Acembly/`
- **ref** RefSeq `http://www.ncbi.nlm.nih.gov/LocusLink/refseq.html`
- **gen** GenScan `http://genes.mit.edu/chris`
- **ens** Ensembl `http://www.ensembl.org`
- **uni** UniGene `http://www.ncbi.nlm.nih.gov/UniGene/`

Gene or Expression Density The number of genes or expressed genes per base pair in the region surrounding the integration site. This is abbreviated as `gene.density` in some displays. There are gene density measures for each of the annotation schemes listed above. The width of the region varies from 100,000 base pairs ("100k") up to 2,000,000 base pairs ("2M") for genes. The expression measures use regions ranging from 25,000 to 32,000,000 base pairs. The expression measures are

- **dens** The density of probesets represented on the Affymetrix HU133a GeneChip.
- **low.ex** The density of probesets achieving the 50th percentile of expression
- **med.ex** The density of probesets achieving the 75th percentile of expression
- **hi.ex** The density of probesets achieving the 87.5^{th} percentile of expression

Dnase I Site Density The number or density of DNAse I sites in regions surrounding the integration (or control) site. This is abbreviated as `dnase` in some displays.

GC Content and CpG Islands The GC percent in the 5kb region containing the site (`gcpct`), whether the site is in a CpG island (`is.cpg`), the number (or density) of CpG islands in the region surrounding the site. This is abbreviated as `cpg` in some displays.

Transcription Start/Stop Features The relation of the site to transcription start/stop position on the same strand using each of the annotation schemes listed above. This is abbreviated as `juxtapos` in some displays. The features are:

start.dx distance to the nearest start position

boundary.dx distance to the nearest start or stop position

general.wd distance from the last start or stop position to the next.

signed.dx the estimated log-odds of integration (vs control) site based on the signed distance to the nearest start site (negative values for upstream, positive for downstream). Previous studies noted strongly non-monotonic effects of the signed distance in MLV integration complexes. To acomodate this, a fitted value was computed as follows: The ranks of the signed distance were found and scaled to range from -1 to 1. The rank that would correspond to the distance zero (i.e. the start site) was noted. A cubic spline logistic (for random controls) or conditional logit (for matched controls) regression is fitted to these scaled ranks using one interior knot located at the rank corresponding to distance zero. In order to avoid overfitting and resubstitution bias in estimates of association with integration, a 10-fold crossvalidation was performed with the log-odds estimated by fitting the model to each training sample and estimating the odds for members of the corresponding test set.

TRANSFAC scores The scores from each of a selection of the position weight matrices (PWMs) for transcription factor binding sites. This is abbreviated as `transfac` in some displays.

Positional Weight in Flanking Sequence The loglikelihood for integration versus control site at each position in twenty bases of flanking sequence (10 upstream and 10 downstream) and their sum. In order to avoid overfitting and resubstitution bias in estimates of association with integration, the score based on the sum of all twenty bases was computed using leave-one-out crossvalidation (which is easily computed for this measure). (The bias of the single base scores is negligible, so direct computation was used.) This is abbreviated as `score.20` in some displays. Further, rather than use the relative frequency estimator for the proportions, the standard Bayes estimator or multinomial proportions based on the no-information uniform prior is used to estimate the "background" base proportions (for discussion of this and related estimators, see [Jones and Vines, 1998]). The proportions for the bases surrounding the integrants are estimated using the background frequencies times 4 as the parameters of a Dirichlet prior, i.e. $\hat{p}_{position,base} = \frac{n_{position,base}+4\hat{\pi}_{base}}{N+4}$, where $n_{position,base}$ is the count of sites with that base in that position relative to the integration site, N is the number of sites, and $\hat{\pi}_{base}$ is the estimate of the "background" proportion for that base.

3 Measuring Association of Features with Integration

A natural measure of the attractiveness of a genomic location (i.e. for a given chromosome, position, and strand) to integration events is the probablility that an integration event would occur there in an experiment in which exactly 1 event occurred. Indexing chromosome by i, position by j, and strand by k and referring to this location in shorthand as "(i, j, k)", this quantity is denoted by

$$\Pr(\text{integration at } (i, j, k)) = \lambda_{ijk}$$

In an experiment in which N independent integration events occurred, the data may be represented by the count of integration events occurring at each site, n_{ijk}. The expected number of events at (i, j, k) is

$$E(n_{ijk}) = N\lambda_{ijk}$$

Associated with each location is a vector of genomic 'features', X_{ijk}, that may include indicators for factors such as whether the location resides in a gene or in an exon, quantitative values such as the GC content of a defined region surrounding the location, and functions of simpler features such as polynomials and cross-products. For a given experiment, the probability that a single integration event occurs at location (i, j, k) may be modelled as

$$\log(\lambda_{ijk}) = \alpha + X_{ijk}\beta$$

Where β is a vector of coefficients that determine the effects of the genomic features on the probability of integration and α is a normalizing constant. The use of such *log-linear models* to study counts is well established in statistics (see [Bishop et al., 1975] and is often approached via *generalized linear models* (or GLMs) using a Poisson link (see [McCullagh and Nelder, 1999]). Although well grounded in both theory and practice, the computational demands of this approach are excessive when one is considering a collection of 6 billion counts as for the human genome.

Fortunately, the parameters of interest, β, can be be estimated by computationally feasible, alternative methods that use well-known relationships between log-linear, logistic, and conditional logit models; these methods involve drawing random samples of locations from the genome and finding logistic (or conditional logit) functions of the genomic features that discriminate between integration events and random samples. The strategy is essentially that of the *nested case-control* study (for a review see [Breslow, 1996]) in which all genomic locations constitute the "cohort", the integration sites are the "cases" or "events", and the randomly sampled genomic locations are the "nested controls".

The equivalence of the regression coefficients estimated in the nested case-control framework to the coefficients of interest, β, is illustrated here briefly. If one genomic location is sampled so that the probability of sampling location (i, j, k) is $\tau_{ijk} = \exp(\nu + X_{ijk}\gamma)$ then the probability that an event found at

(i, j, k) is the actual integration event when one integration and one control site have been sampled is

$$\Pr\left((i, j, k) \text{ is integration site}\right) = \frac{\exp(\alpha + X_{ijk}\beta)}{\exp(\alpha + X_{ijk}\beta) + \exp(\nu + X_{ijk}\gamma)}$$

If all genomic locations are sampled with equal probability then $\gamma = 0$ and the probability that an event found at (i, j, k) is the actual integration event when one integration and one control site have been sampled is

$$\Pr\left((i, j, k) \text{ is integration site}\right) = \frac{\exp(\tilde{\alpha} + X_{ijk}\beta)}{1 + \exp(\tilde{\alpha} + X_{ijk}\beta)}$$

where $\tilde{\alpha} = \alpha - \nu$. The log-odds that the event at (i, j, k) is the actual integration event is

$$\log\left(\frac{\Pr\left((i, j, k) \text{ is integration site}\right)}{1 - \Pr\left((i, j, k) \text{ is integration site}\right)}\right) = \tilde{\alpha} + X_{ijk}\beta$$

The linear logistic form used here is the basis for logistic regression analysis. To this point, only one integration event and one control event have been considered. When N integration and M control events are sampled, similar expressions are obtained but the coefficient corresponding to α increases by $\log(N)$, that for ν increases by $\log(M)$, and and that for $\tilde{\alpha}$ changes by $\log(N/M)$.

The regression coefficients β have useful interpretations. When X_{ijk} is a single binary feature (e.g. for being in a gene or not being in a gene) coded as one or zero (e.g. for 'in a gene' or 'not in a gene'), the regression coefficient β estimates the difference in the log-odds associated with that feature in the context of a choice between actual and randomly sampled integration events. When a quantitative feature is used (e.g. the number of genes within 1 megabase of (i, j, k)), the importance of a given value of the coefficient estimates the difference in the log-odds for a one unit increase in the quantitative feature (e.g. the difference due to having one more gene within 1 megabase of (i, j, k)). In the context of modelling $N\lambda_{ijk}$, the expected number of integration events of integration at a particular location given N events, $exp(\beta)$ is the factor by which the number of integration events increases due to the binary feature or due to a one unit increase in a quantitative feature. Thus, the coefficients of these models provide a basis for assessing the importance of a genomic feature on integration or comparing the impacts of different features.

More general machine learning algorithms are available that do not require a pre-specified form for the candidate features. Typically, the output of these machine learners is an algorithm that predicts into which group a new observation should be classified. In some cases, a measure of certainty for the group assignment (eg. posterior probability) is provided. Machine learning algorithms such as random Forest ^{TM}[Breiman, 2001] offer greater flexibility than regression model that require an explicit functional form for the relationships between predictor variables and the predicted category. However, when the form of the regression model is a fair approximation to the optimal predictor, it will perform much better when trained with small datasets.

A commonly used measure of a predictor variable's ability to discriminate between two classes of events is the area under the Receiver Operator Characteristic (ROC) curve. The ROC curve plots the true positive rate (i.e. the fraction of integration events above above a fixed cutpoint) on the vertical axis versus the false positive rate (i.e. the fraction of integration events above that same cutpoint) for all possible cutpoints. The area under the curve is 1.0 when all integration events have higher values for the feature than any control event and 0.0 for the opposite case. When the area is 0.5 it is equally likely that either has a higher value or that the two are tied. Two competing predictors can be compared by assessing the area under the ROC curve for each of them. The ROC curve area, z, for classifying an event as an integration or control event based on a quantity x_{ijk} that represents a genomic feature at each location (i, j, k) can be computed as

$$z = \sum_{ijk} (\lambda_{ijk}(\#(rst : x_{ijk} > x_{rst}) + \#(rst : x_{ijk} = x_{rst})/2)/L)$$

where L is the number of genomic locations. So, the area under the ROC Curve provides a summary of the impact of the feature on integration intensity.

It may be important to distinguish between events occurring and events being recovered. Some methods for recovering integration sites have a known bias (i.e. some methods tend to recover more events near — but not too near — specific restriction sites). Suppose the recovery rate at location (i, j, k) is given by μ_{ijk}, then

$$\tilde{\lambda}_{ijk} = \frac{\lambda_{ijk}\mu_{ijk}}{\sum_{rst} \lambda_{rst}\mu_{rst}}$$

is the expected number of sites recovered at (i, j, k) when 1 is recovered somewhere. Often, it is possible to mimic that bias in sampling from the genome. In that case,

$$\tilde{\tau}_{ijk} = \frac{\tau_{ijk}\mu_{ijk}}{\sum_{rst} \tau_{rst}\mu_{rst}}$$

is the number of events expected at (i, j, k) when 1 is sampled. Given that the control events are drawn with a similar bias, then

$$\tilde{\phi}_{ijk} = \frac{\tilde{\lambda}_{ijk}}{\tilde{\tau}_{ijk}} = \phi_{ijk}\rho$$

where $\rho = \sum_{rst} \lambda_{rst}\mu_{rst} / \sum_{rst} \tau_{rst}\mu_{rst}$. So, regression models for $\log \tilde{\phi}$ will differ from those for $\log \phi$ by a constant. Conclusions concerning the effects of genomic features are unaffected by biased sampling so long as control sites sampled with the same bias are matched to each integration site. When conditional logit regression models are used to fit data in which control sites are matched according to the distance from the corresponding restriction site, constants like ρ drop out. ROC curve areas for such data will be computed conditionally on the matched controls, i.e. the area reflects the proportion of sites whose predicted value exceeds those of its matched controls.

4 Association of Features with Integration Targetting

In this section the association of each of the features with integration targetting is described. The area under the ROC curve for predicting integration vs control targetting is taken as the measure of association. This measure can be interpreted as the probability that a randomly drawn integration site will have a value for its genomic feature that exceeds that of a random (or matched) control. Thus, values very near 0.50 are consistent with having no predictive value, values very near 1.0 occur when higher values of the feature predict integration, and values very near 0.0 occur when lower values of the feature predict integration.

Two of the features are derived from fitting the data at hand. Ordinarily, this would generate a bias in assessing the usefulness of those derived features. To obviate that bias, cross-validation procedures are applied for the `score.20` and `signed.dx` features. These procedures were described earlier.

Since there are a several hundred features and 17 datasets, a compact representation of these associations is needed. An overview is given by a boxplot of the improvement over chance performance as measured by the area under the ROC curve; this improvement is the absolute difference between the area and 0.50. Values around 0.0 indicate no useful predictive information in the feature, while values near 0.50 indicate that the feature is nearly perfect in separating integration sites from random or matched controls. Each box indicates the first and third quartiles of the values, while heavy line in the middle gives the median value. The 'whiskers' extend to the most extreme observation within 1.5 times the interquartile range of the median. Individual points beyond the whiskers are plotted separately. More detailed results are given later by using a false color map to display the matrix of associations for each type of genomic feature using rows of the matrix for features and columns for data sets. Here the results are displayed according to the groupings of features described above. The overall score for the positional weight matrix of the 20 bases flanking the integration site (`score.20.all`) is presented separately from the 20 scores for each base pair (`score.20.1.bp`).

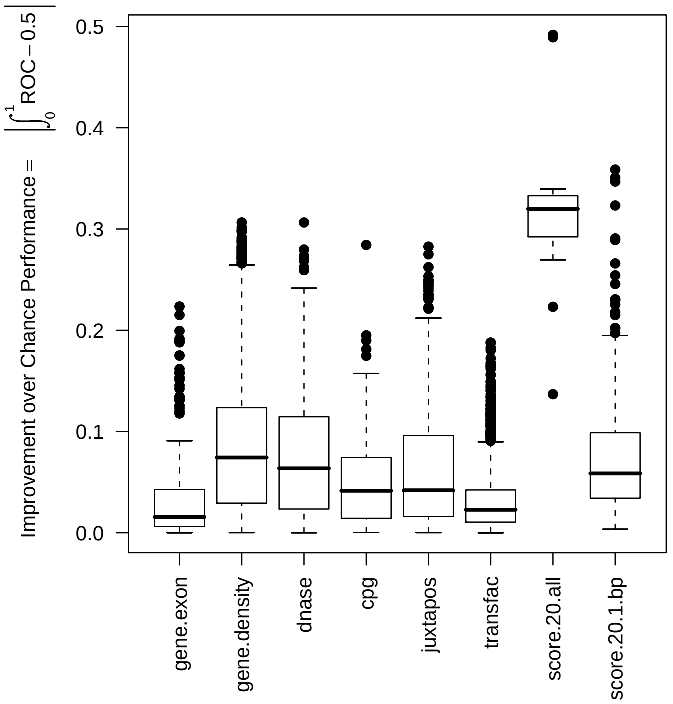

As is evident, the overall score for the positional weight matrix of the 20 bases flanking the integration site yields nearly perfect prediction in two integration complexes. The median value exceeds the best values of all of the features in the other categories. Even the single base pair scores do well in comparison to most of the other feature categories.

To get a more detailed view of these results, false color maps display the matrices of associations for each type of genomic feature using rows of the matrix for features and columns for data sets. Black represents ROC curve areas of 0.50 (no association), bright green corresponds to areas near zero, while bright red corresponds to values near one as shown in this figure:

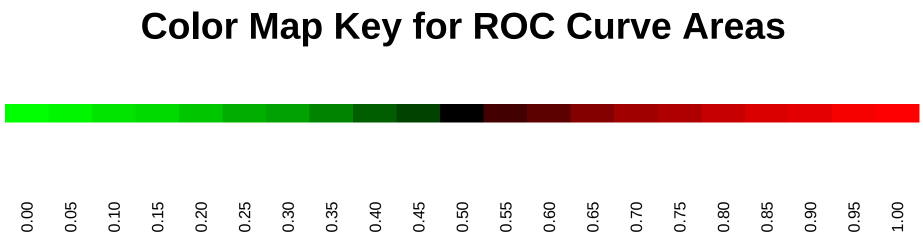

In addition for each graph, a table is presented that breaks down the ROC curve areas into several categories and counts the number of features that fall into each category for each data set.

4.1 Gene or Exon

The distribution of ROC curve areas is given in this table:

	(0,0.2]	(0.2,0.3]	(0.3,0.4]	(0.4,0.6]	(0.6,0.7]	(0.7,0.8]	(0.8,1]
AAV-Fibro	0	0	0	10	0	0	0
ASLV-HeLa	0	0	0	10	0	0	0
ASLV-293T	0	0	0	10	0	0	0
HIV-Mac	0	0	0	6	4	0	0
HIV-SupT1	0	0	0	6	4	0	0
HIV-293T	0	0	0	6	4	0	0
HIV-Jurkat	0	0	0	6	2	2	0
HIV-IMR90	0	0	0	6	4	0	0
HIV-PBMC	0	0	0	6	4	0	0
L1-Hela	0	0	0	10	0	0	0
L1-Hela/HCT	0	0	0	10	0	0	0
MLV-HeLa-S	0	0	0	10	0	0	0
MLV-HeLa-NS	0	0	0	10	0	0	0
SFV-CD34+	0	0	0	10	0	0	0
SFV-Fibro	0	0	0	10	0	0	0
SB-Hela	0	0	0	10	0	0	0
SB-Huh-7	0	0	0	10	0	0	0

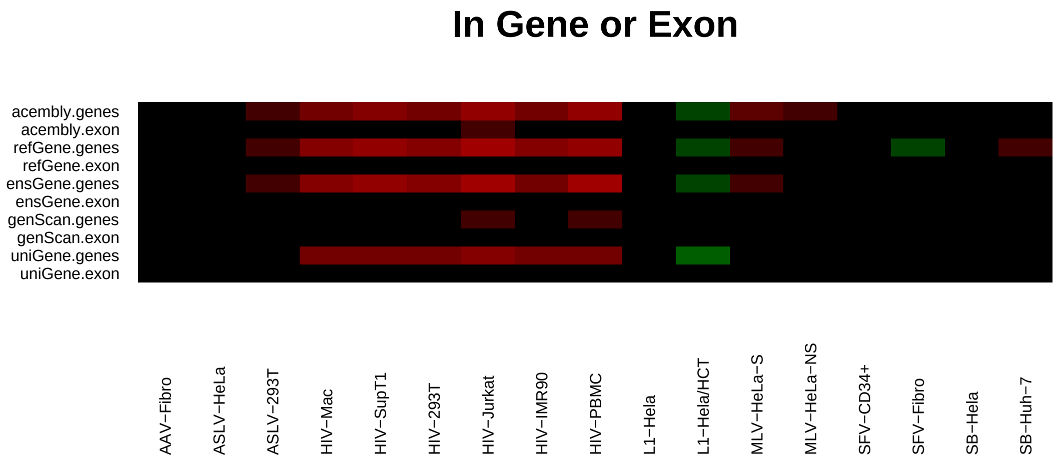

4.2 Gene or Expression Density

The distribution of ROC curve areas is given in this table:

	(0,0.2]	(0.2,0.3]	(0.3,0.4]	(0.4,0.6]	(0.6,0.7]	(0.7,0.8]	(0.8,1]
AAV-Fibro	0	0	0	69	0	0	0
ASLV-HeLa	0	0	0	54	15	0	0
ASLV-293T	0	0	0	45	24	0	0
HIV-Mac	0	0	0	52	17	0	0
HIV-SupT1	0	0	0	11	22	36	0
HIV-293T	0	0	0	12	55	2	0
HIV-Jurkat	0	0	0	4	22	42	1
HIV-IMR90	0	0	0	40	29	0	0
HIV-PBMC	0	0	0	36	29	4	0
L1-Hela	0	0	0	57	12	0	0
L1-Hela/HCT	0	0	0	68	1	0	0
MLV-HeLa-S	0	0	0	7	14	47	1
MLV-HeLa-NS	0	0	0	25	44	0	0
SFV-CD34+	0	0	0	63	6	0	0
SFV-Fibro	0	0	0	69	0	0	0
SB-Hela	0	0	0	69	0	0	0
SB-Huh-7	0	0	0	68	1	0	0

Gene or Expression Density

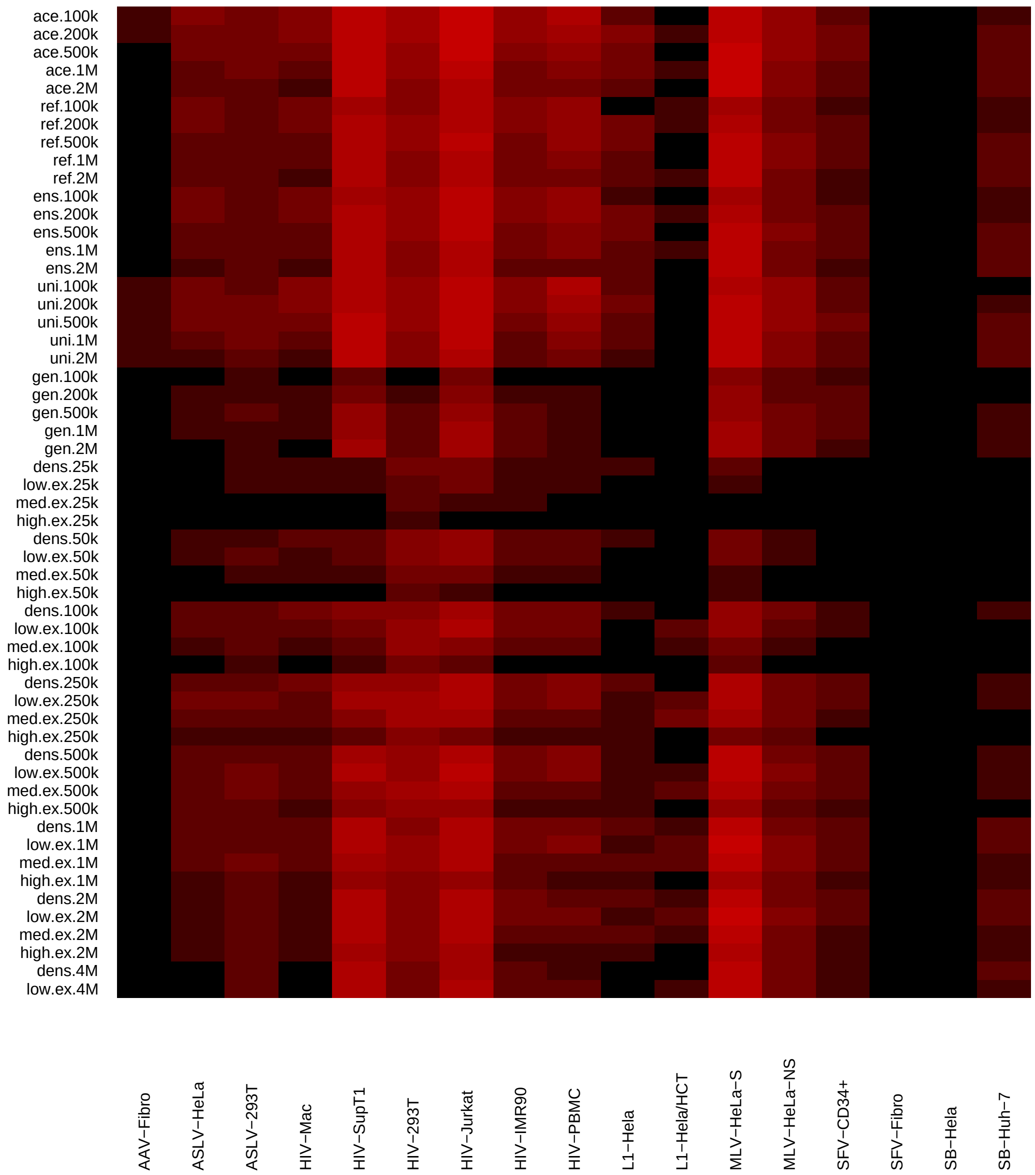

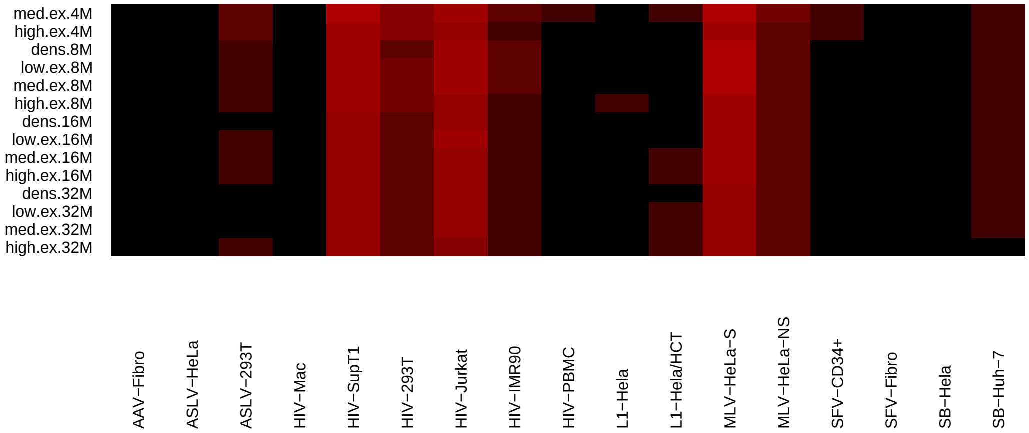
med.ex.4M
high.ex.4M
dens.8M
low.ex.8M
med.ex.8M
high.ex.8M
dens.16M
low.ex.16M
med.ex.16M
high.ex.16M
dens.32M
low.ex.32M
med.ex.32M
high.ex.32M
AAV−Fibro
ASLV−HeLa
ASLV−293T
HIV−Mac
HIV−SupT1
HIV−293T
HIV−Jurkat
HIV−IMR90
HIV−PBMC
L1−Hela
L1−Hela/HCT
MLV−HeLa−S
MLV−HeLa−NS
SFV−CD34+
SFV−Fibro
SB−Hela
SB−Huh−7

4.3 Dnase I Site Density

The distribution of ROC curve areas is given in this table:

	(0,0.2]	(0.2,0.3]	(0.3,0.4]	(0.4,0.6]	(0.6,0.7]	(0.7,0.8]	(0.8,1]
AAV-Fibro	0	0	0	9	0	0	0
ASLV-HeLa	0	0	0	8	1	0	0
ASLV-293T	0	0	0	6	3	0	0
HIV-Mac	0	0	0	8	1	0	0
HIV-SupT1	0	0	0	3	2	4	0
HIV-293T	0	0	0	5	4	0	0
HIV-Jurkat	0	0	0	2	2	5	0
HIV-IMR90	0	0	0	6	3	0	0
HIV-PBMC	0	0	0	5	3	1	0
L1-Hela	0	0	0	8	1	0	0
L1-Hela/HCT	0	0	0	9	0	0	0
MLV-HeLa-S	0	0	0	1	2	5	1
MLV-HeLa-NS	0	0	0	3	6	0	0
SFV-CD34+	0	0	0	5	4	0	0
SFV-Fibro	0	0	0	9	0	0	0
SB-Hela	0	0	0	9	0	0	0
SB-Huh-7	0	0	0	8	1	0	0

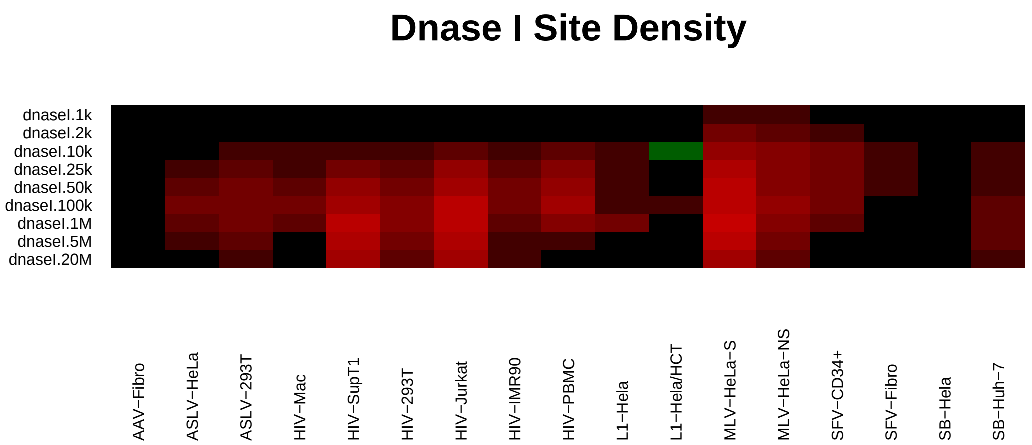

4.4 GC Content and CpG Islands

The distribution of ROC curve areas is given in this table:

	(0,0.2]	(0.2,0.3]	(0.3,0.4]	(0.4,0.6]	(0.6,0.7]	(0.7,0.8]	(0.8,1]
AAV-Fibro	0	0	0	18	0	0	0
ASLV-HeLa	0	0	0	18	0	0	0
ASLV-293T	0	0	0	18	0	0	0
HIV-Mac	0	0	0	18	0	0	0
HIV-SupT1	0	0	0	12	6	0	0
HIV-293T	0	0	0	16	2	0	0
HIV-Jurkat	0	0	0	10	8	0	0
HIV-IMR90	0	0	0	18	0	0	0
HIV-PBMC	0	0	0	17	1	0	0
L1-Hela	0	0	3	14	1	0	0
L1-Hela/HCT	0	0	0	18	0	0	0
MLV-HeLa-S	0	0	0	7	10	1	0
MLV-HeLa-NS	0	0	0	13	5	0	0
SFV-CD34+	0	0	0	15	3	0	0
SFV-Fibro	0	0	0	18	0	0	0
SB-Hela	0	0	0	18	0	0	0
SB-Huh-7	0	0	0	17	1	0	0

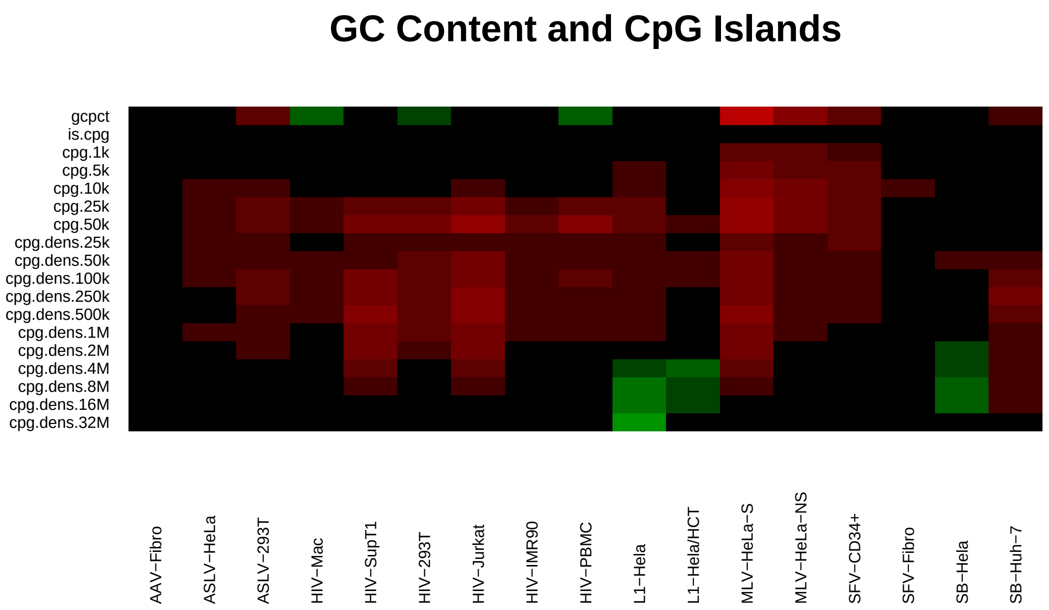

4.5 Transcription Start/Stop Features

The distribution of ROC curve areas is given in this table:

	(0,0.2]	(0.2,0.3]	(0.3,0.4]	(0.4,0.6]	(0.6,0.7]	(0.7,0.8]	(0.8,1]
AAV-Fibro	0	0	0	20	0	0	0
ASLV-HeLa	0	0	1	19	0	0	0
ASLV-293T	0	0	1	19	0	0	0
HIV-Mac	0	0	2	14	4	0	0
HIV-SupT1	0	1	3	12	1	3	0
HIV-293T	0	0	3	13	3	1	0
HIV-Jurkat	0	2	2	12	0	4	0
HIV-IMR90	0	0	3	13	4	0	0
HIV-PBMC	0	1	3	12	0	4	0
L1-Hela	0	1	5	13	1	0	0
L1-Hela/HCT	0	0	1	19	0	0	0
MLV-HeLa-S	0	3	3	9	2	3	0
MLV-HeLa-NS	0	0	4	12	4	0	0
SFV-CD34+	0	0	4	13	3	0	0
SFV-Fibro	0	0	0	20	0	0	0
SB-Hela	0	0	0	20	0	0	0
SB-Huh-7	0	0	0	20	0	0	0

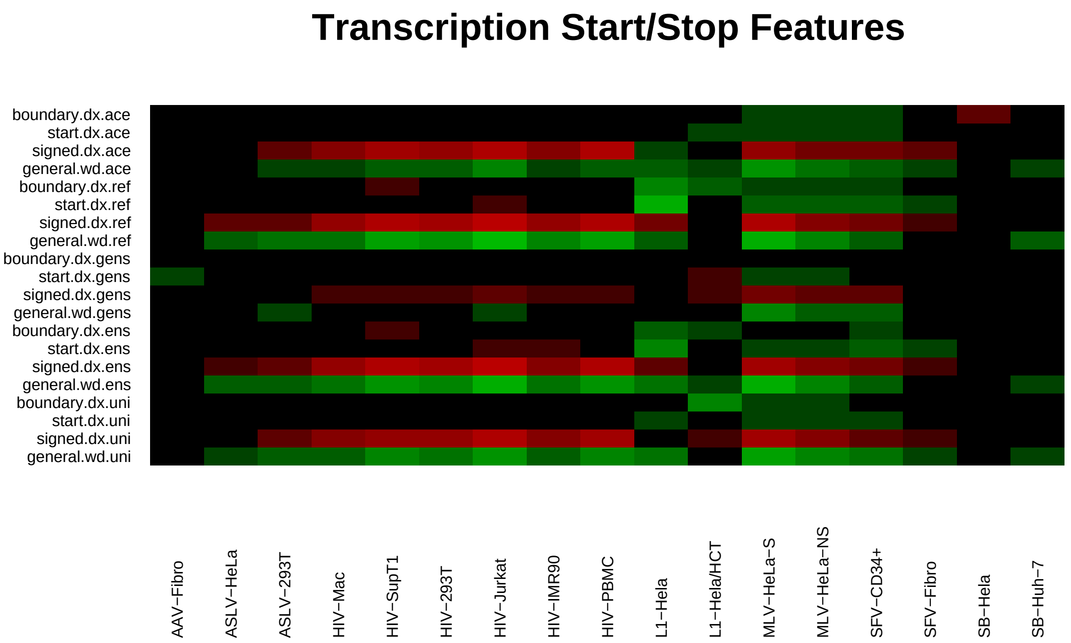

4.6 TRANSFAC scores

The distribution of ROC curve areas is given in this table:

	(0,0.2]	(0.2,0.3]	(0.3,0.4]	(0.4,0.6]	(0.6,0.7]	(0.7,0.8]	(0.8,1]
AAV-Fibro	0	0	0	110	0	0	0
ASLV-HeLa	0	0	0	110	0	0	0
ASLV-293T	0	0	0	110	0	0	0
HIV-Mac	0	0	0	110	0	0	0
HIV-SupT1	0	0	0	105	5	0	0
HIV-293T	0	0	0	110	0	0	0
HIV-Jurkat	0	0	0	110	0	0	0
HIV-IMR90	0	0	0	110	0	0	0
HIV-PBMC	0	0	0	110	0	0	0
L1-Hela	0	0	2	108	0	0	0
L1-Hela/HCT	0	0	5	103	2	0	0
MLV-HeLa-S	0	0	5	70	35	0	0
MLV-HeLa-NS	0	0	0	97	13	0	0
SFV-CD34+	0	0	0	110	0	0	0
SFV-Fibro	0	0	0	110	0	0	0
SB-Hela	0	0	0	110	0	0	0
SB-Huh-7	0	0	0	110	0	0	0

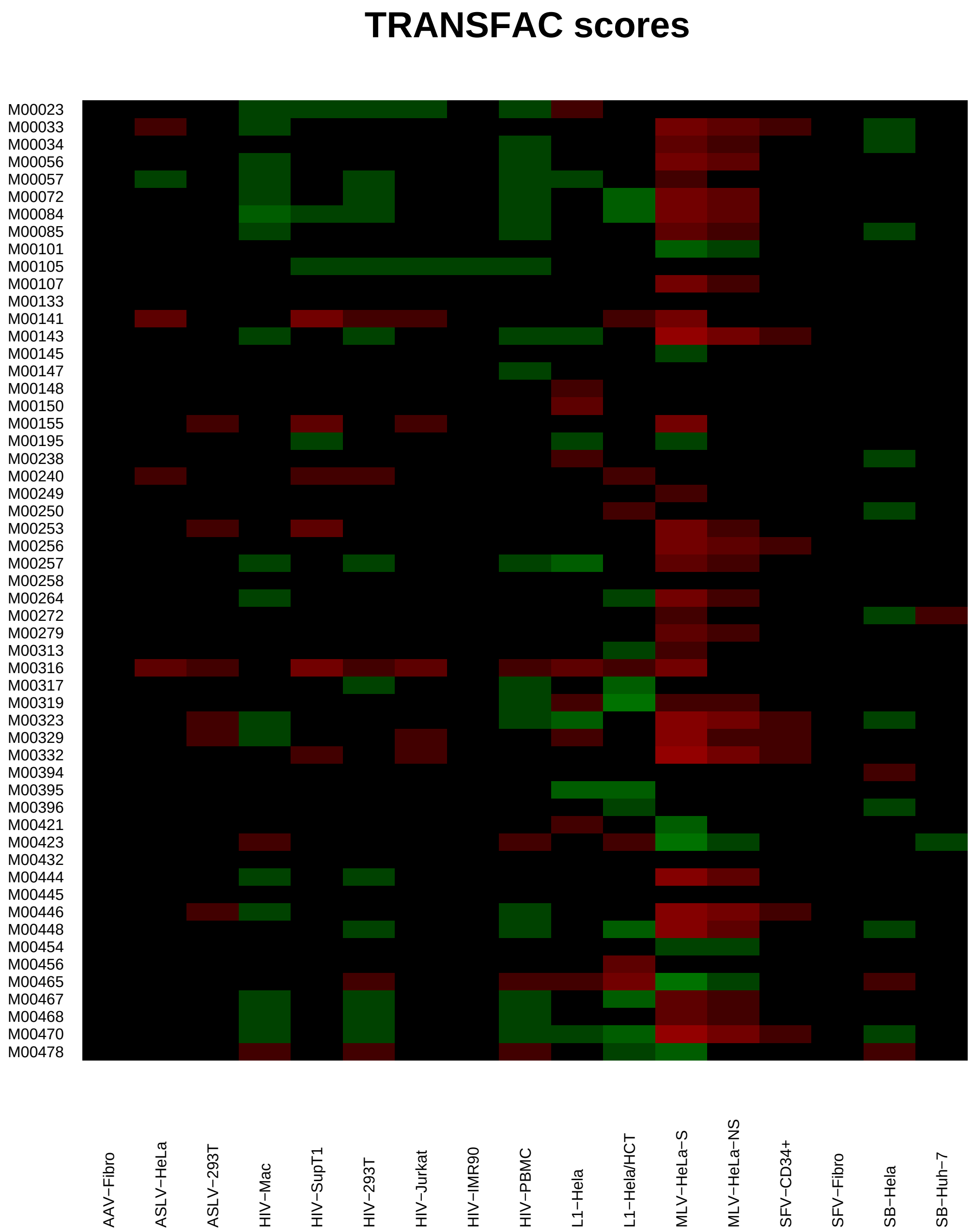
TRANSFAC scores
M00023
M00033
M00034
M00056
M00057
M00072
M00084
M00085
M00101
M00105
M00107
M00133
M00141
M00143
M00145
M00147
M00148
M00150
M00155
M00195
M00238
M00240
M00249
M00250
M00253
M00256
M00257
M00258
M00264
M00272
M00279
M00313
M00316
M00317
M00319
M00323
M00329
M00332
M00394
M00395
M00396
M00421
M00423
M00432
M00444
M00445
M00446
M00448
M00454
M00456
M00465
M00467
M00468
M00470
M00478
AAV−Fibro
ASLV−HeLa
ASLV−293T
HIV−Mac
HIV−SupT1
HIV−293T
HIV−Jurkat
HIV−IMR90
HIV−PBMC
L1−Hela
L1−Hela/HCT
MLV−HeLa−S
MLV−HeLa−NS
SFV−CD34+
SFV−Fibro
SB−Hela
SB−Huh−7

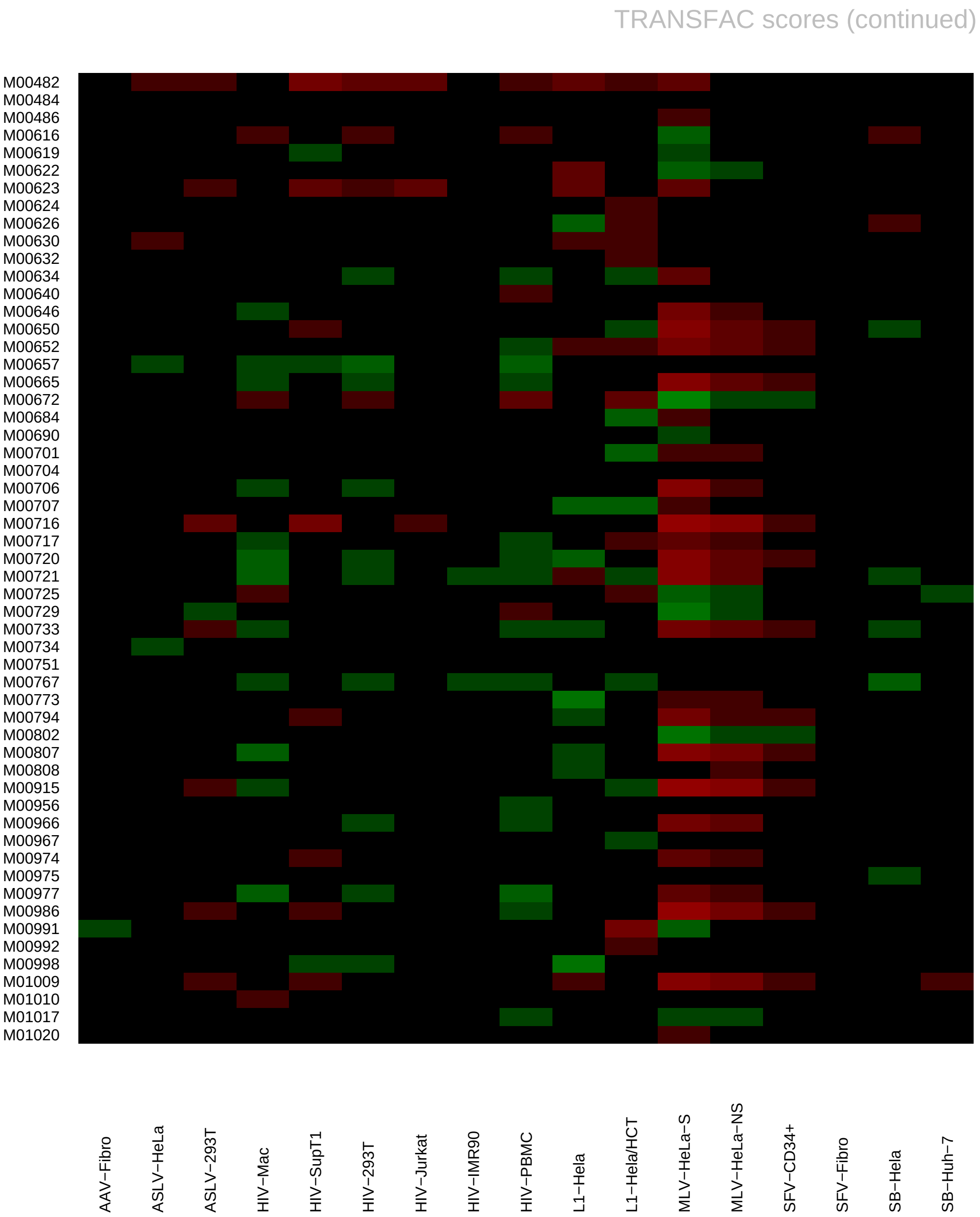
TRANSFAC scores (continued)
M00482
M00484
M00486
M00616
M00619
M00622
M00623
M00624
M00626
M00630
M00632
M00634
M00640
M00646
M00650
M00652
M00657
M00665
M00672
M00684
M00690
M00701
M00704
M00706
M00707
M00716
M00717
M00720
M00721
M00725
M00729
M00733
M00734
M00751
M00767
M00773
M00794
M00802
M00807
M00808
M00915
M00956
M00966
M00967
M00974
M00975
M00977
M00986
M00991
M00992
M00998
M01009
M01010
M01017
M01020
AAV−Fibro
ASLV−HeLa
ASLV−293T
HIV−Mac
HIV−SupT1
HIV−293T
HIV−Jurkat
HIV−IMR90
HIV−PBMC
L1−Hela
L1−Hela/HCT
MLV−HeLa−S
MLV−HeLa−NS
SFV−CD34+
SFV−Fibro
SB−Hela
SB−Huh−7

4.7 Positional Weight in Flanking Sequence

The distribution of ROC curve areas is given in this table:

	(0,0.2]	(0.2,0.3]	(0.3,0.4]	(0.4,0.6]	(0.6,0.7]	(0.7,0.8]	(0.8,1]
AAV-Fibro	0	0	0	19	2	0	0
ASLV-HeLa	0	0	0	18	2	1	0
ASLV-293T	0	0	0	17	3	1	0
HIV-Mac	0	0	0	15	5	0	1
HIV-SupT1	0	0	0	17	3	0	1
HIV-293T	0	0	0	15	5	0	1
HIV-Jurkat	0	0	0	18	2	0	1
HIV-IMR90	0	0	0	15	5	0	1
HIV-PBMC	0	0	0	13	7	0	1
L1-Hela	0	0	0	5	8	5	3
L1-Hela/HCT	0	0	0	9	8	3	1
MLV-HeLa-S	0	0	0	16	4	0	1
MLV-HeLa-NS	0	0	0	17	3	1	0
SFV-CD34+	0	0	0	18	1	1	1
SFV-Fibro	0	0	0	18	2	1	0
SB-Hela	0	0	0	14	4	2	1
SB-Huh-7	0	0	0	13	3	2	3

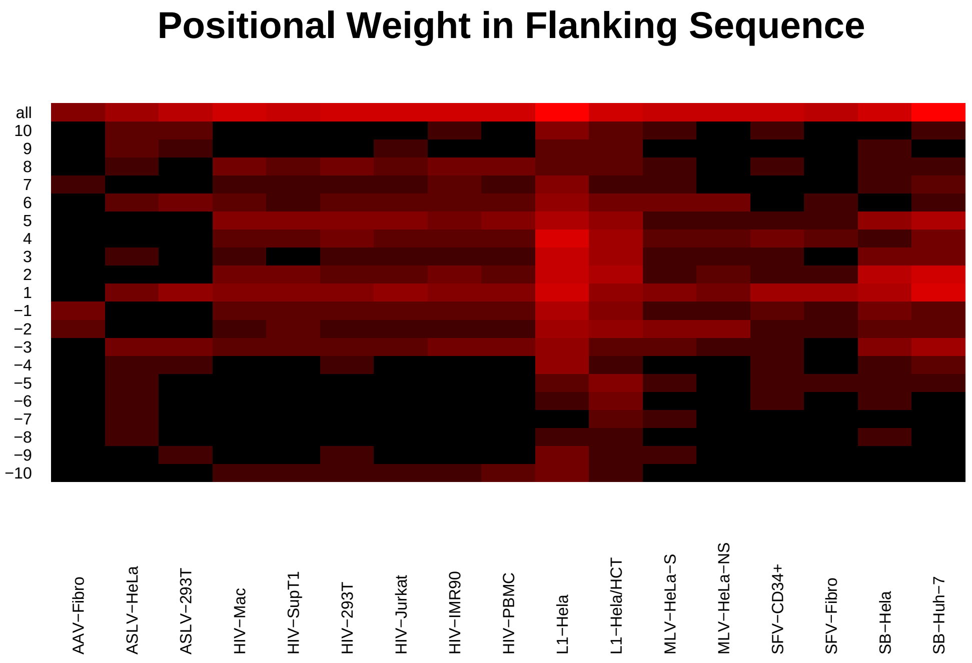

5 Details of Local Sequence Effects

The strongest association for each integration complex was the score based on 20 base pairs that flank the integration site. In some cases, near perfect discrimination (ROC area greater than 0.98) is possible. Given this some deeper exploration into which motifs are associated with integration seems in order. First, here is a display of the weights used in the position weight matrix (PWM) scoring. The score is obtained by adding the value read on the y axis for each base seen according to the position indicated on the x axis. Positions with negative numbers indicate the number of bases 'upstream' of the integration site in the direction of transcription, while those with positive numbers indicate the number of bases 'downstream' of the integration site. When the oligonucleotide base appears above the horizontal line the site is more attractive to the integration complex and when it appears below the line it is less attractive. When viewing these with a pdf viewer, it helps to enlarge the zoom in on the image.

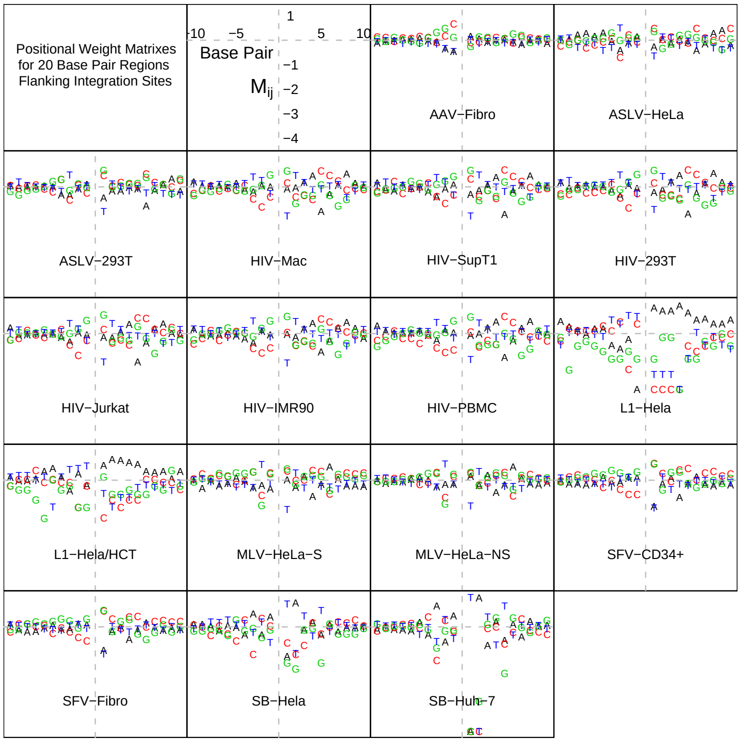

Similar motifs appear in several of the integration complexes. To make it easier to determine the similarity of scores for the different integration complexes, a sample of random genomic sites is scored using each of the PWMs and the correlations of those scores are displayed in false color in the following figure. Green corresponds to negative correlations (meaning that when one integration site is favored the other integration complex tends to be disfavored) and red corresponds to positive correlations (both complexes tend to be favored

or disfavored in tandem). The integration complexes are subjected to hierarchical clustering of the correlations, which is shown on the figure, and ordered according to cluster membership.

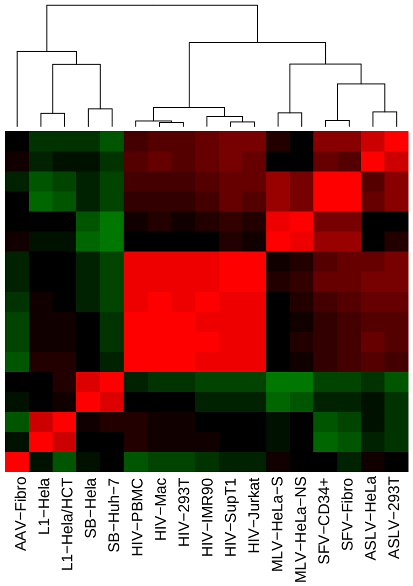

As is evident, the integration complex scores form distinct groups. Looking back to the earlier figure, the similarites in PWMs that account for these correlations are usually apparent. The HIV integration complexes all cluster together, and favoring G and disfavoring T bases at position 1 and favoring C and disfavoring A at position 5. Many other effects are evident both upstream and downstream in the HIV complexes. The L1-Hela and L1-Hela/HCT complex scores favor A downstream. The SB-Hela and SB-Huh-7 scores strongly favor TA immediate downstream of the integration site. The SFV complexes prefer G or C immediately downstream, and weakly favor C further downstream in several positions. The ASLV and SFV scores tend to correlate weakly with the HIV scores and similarites at positions -3, 1, and 5 are apparent.

6 Association of Local Sequence and Other Features

Given that the score for the 20bp flanking region (“`score.20`”) showed the strongest association with integration targetting, it is worth considering whether

the other features are merely redundant. One way to do this is to check whether some of those features are highly correlated with `score.20`. Here is a histogram of the correlations of the features with `score.20` for each of the data sets.

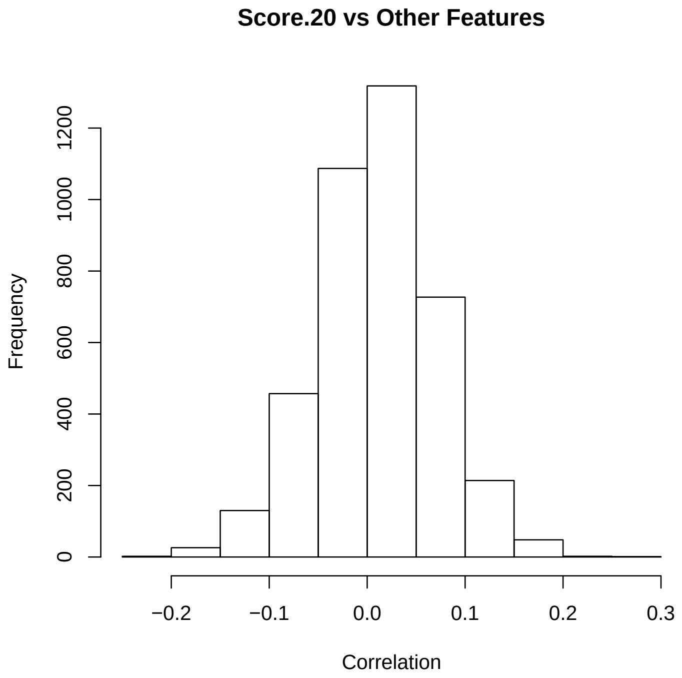

Evidently, the correlations range from -0.218 to 0.263. Most correlations are quite small, so we suspect that there is limited redundancy with the score for the 20bp flanking region. Thus, a predictor of integration targetting constructed from `score.20` and other features could substantially improve upon a score based on `score.20` alone.

7 Incremental Effects of Features

It was obvious above that the local nucleotide sequence is the single most important determinant of integration targetting. However, the median (cross-validated estimate of the) ROC curve area is only 0.82, so there are evidently other factors at play in determining the integration site for most integration complexes.

Here, the incremental effects of the features examined above on the ROC curve area are studied. The method operates as follows: for each feature a (conditional logit or logistic) regression is fit to the data that includes the score for the 20bp flanking region ("`score.20`") and one or more additional terms to represent one of the other features. If the feature is a zero-one indicator (such as `acembly.genes`), then it is included as is. If the feature is a quantitative

measure (such as `ace.100k`), then it is replaced with the basis vectors for a cubic spline of the ranks of the values. In the case of continuous measures, this allows the logarithm of integration intensity to depend on the value of the feature in a (possibly) non-linear fashion. The fitted value of the integration intensity is then use to calculate the under under the ROC curve and the difference of this area minus the area for the curve based only on `score.20` is taken as a measure of the improvement due to the feature. Note that since the criterion optimized by the regressions does not have a one-for-one relation with the area under the ROC curve, it is possible that no improvement or even negative 'improvement' will occur.

A brief overview of the effects of different feature categories in improving prediction is obtained from boxplots of the improvement in area under the ROC curve area when each feature is fitted along with the twenty base pair score.

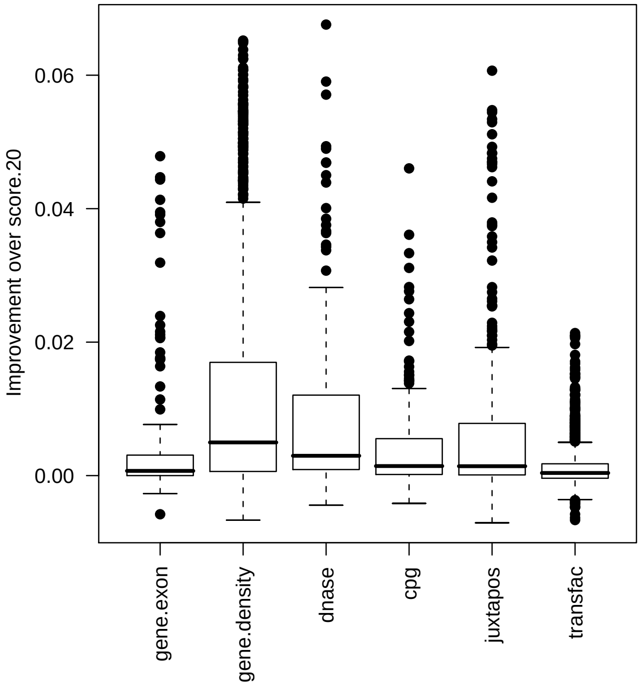

Evidently, there are many features that can improve the prediction of integration targetting by at least a modest amount. Overall, the gene or expression density features seem most promising.

To get a more detailed view, these 'improvement' values are displayed in grayscale with white corresponding to no (or negative) improvement, pure black corresponding to an improvement of 0.10, and shades of gray corresponding to intermediate values.

Here is the color key:

Map Key for Incremental ROC Curve Areas

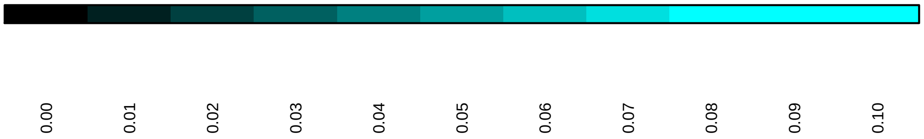

7.1 Gene or Exon

The distribution of ROC curve increments is given in this table:

	≤ 0.01	(0.01,0.02]	(0.02,0.03]	(0.03,0.04]	(0.04,0.05]	>0.05
AAV-Fibro	10	0	0	0	0	0
ASLV-HeLa	10	0	0	0	0	0
ASLV-293T	10	0	0	0	0	0
HIV-Mac	6	1	3	0	0	0
HIV-SupT1	6	1	0	2	1	0
HIV-293T	7	1	2	0	0	0
HIV-Jurkat	6	0	1	1	2	0
HIV-IMR90	6	4	0	0	0	0
HIV-PBMC	6	1	0	2	1	0
L1-Hela	10	0	0	0	0	0
L1-Hela/HCT	10	0	0	0	0	0
MLV-HeLa-S	10	0	0	0	0	0
MLV-HeLa-NS	10	0	0	0	0	0
SFV-CD34+	10	0	0	0	0	0
SFV-Fibro	10	0	0	0	0	0
SB-Hela	10	0	0	0	0	0
SB-Huh-7	10	0	0	0	0	0

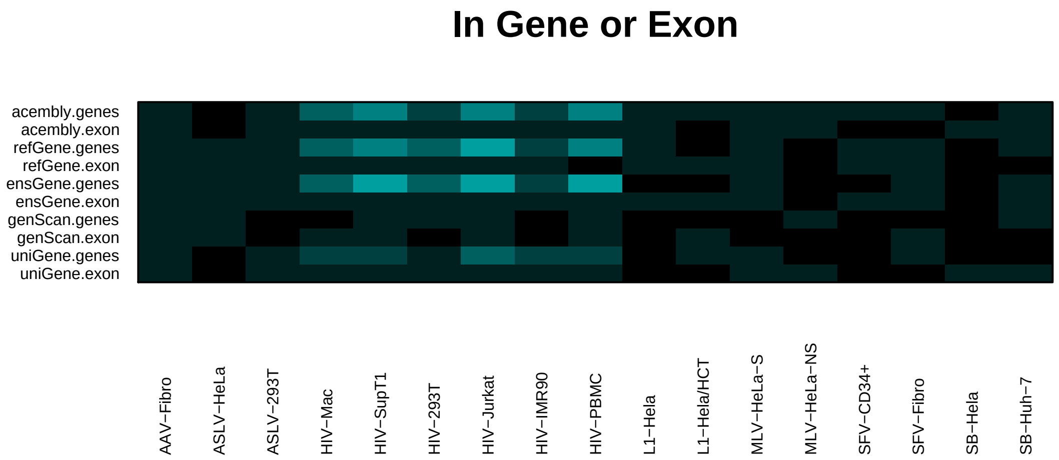

7.2 Gene or Expression Density

The distribution of ROC curve increments is given in this table:

	≤ 0.01	(0.01,0.02]	(0.02,0.03]	(0.03,0.04]	(0.04,0.05]	>0.05
AAV-Fibro	69	0	0	0	0	0
ASLV-HeLa	48	21	0	0	0	0
ASLV-293T	39	30	0	0	0	0
HIV-Mac	44	22	3	0	0	0
HIV-SupT1	5	8	10	16	16	14
HIV-293T	7	17	41	4	0	0
HIV-Jurkat	1	6	12	17	17	16
HIV-IMR90	55	14	0	0	0	0
HIV-PBMC	16	21	14	8	6	4
L1-Hela	69	0	0	0	0	0
L1-Hela/HCT	66	3	0	0	0	0
MLV-HeLa-S	7	7	10	12	12	21
MLV-HeLa-NS	32	32	5	0	0	0
SFV-CD34+	69	0	0	0	0	0
SFV-Fibro	69	0	0	0	0	0
SB-Hela	69	0	0	0	0	0
SB-Huh-7	69	0	0	0	0	0

Gene or Expression Density

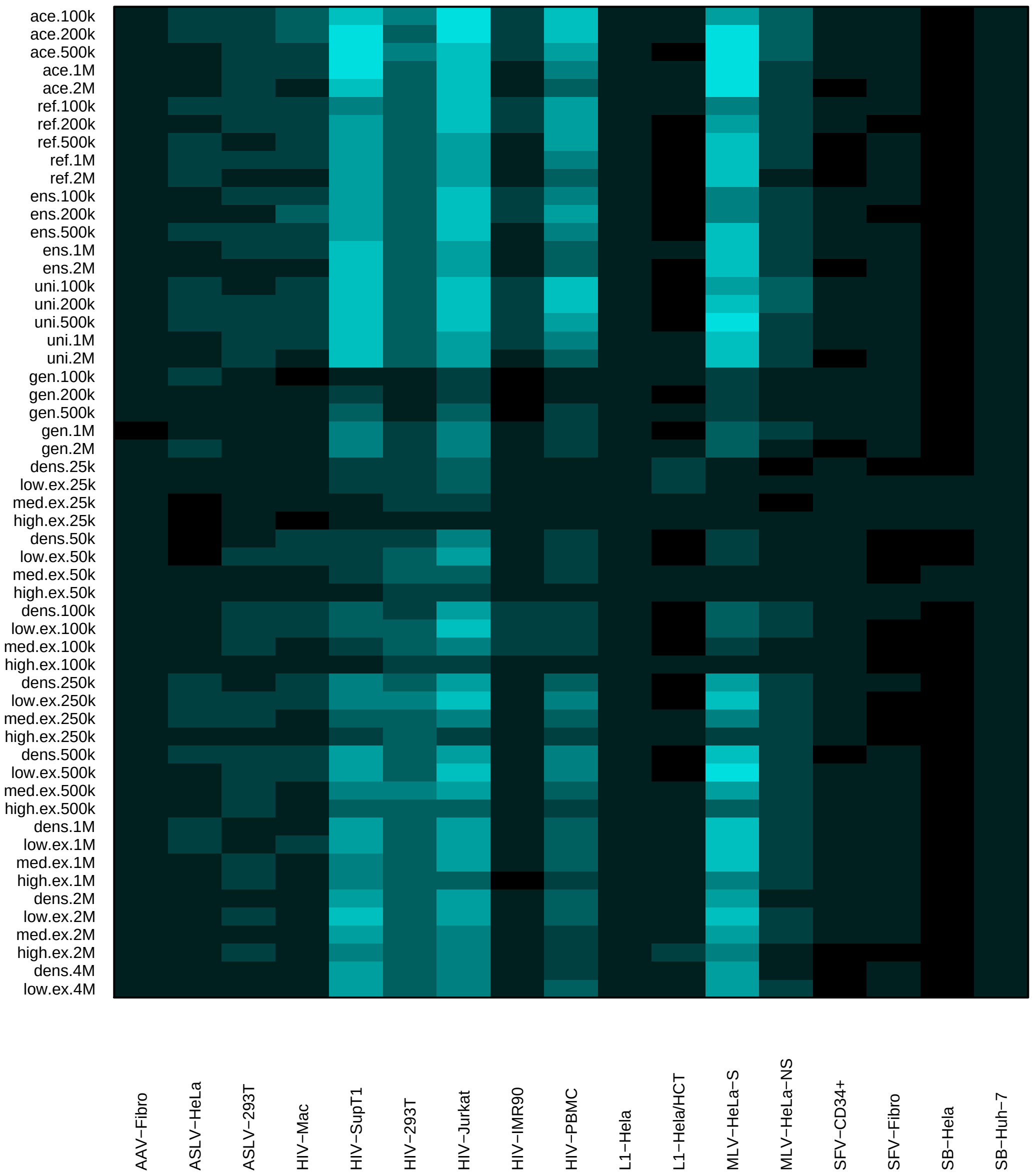

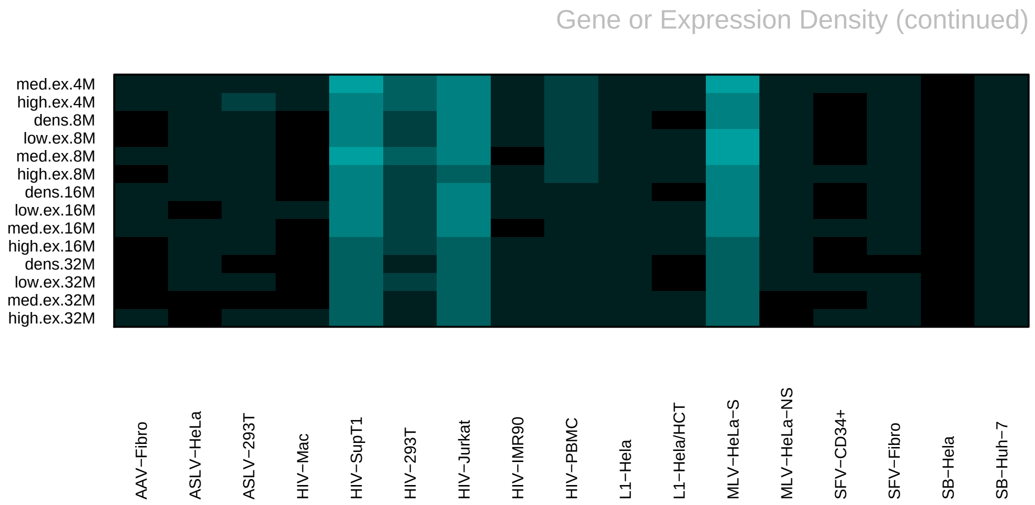

Gene or Expression Density (continued)
med.ex.4M
high.ex.4M
dens.8M
low.ex.8M
med.ex.8M
high.ex.8M
dens.16M
low.ex.16M
med.ex.16M
high.ex.16M
dens.32M
low.ex.32M
med.ex.32M
high.ex.32M
AAV−Fibro
ASLV−HeLa
ASLV−293T
HIV−Mac
HIV−SupT1
HIV−293T
HIV−Jurkat
HIV−IMR90
HIV−PBMC
L1−Hela
L1−Hela/HCT
MLV−HeLa−S
MLV−HeLa−NS
SFV−CD34+
SFV−Fibro
SB−Hela
SB−Huh−7

7.3 Dnase I Site Density

The distribution of ROC curve areas is given in this table:

	≤ 0.01	(0.01,0.02]	(0.02,0.03]	(0.03,0.04]	(0.04,0.05]	>0.05
AAV-Fibro	9	0	0	0	0	0
ASLV-HeLa	7	2	0	0	0	0
ASLV-293T	5	4	0	0	0	0
HIV-Mac	9	0	0	0	0	0
HIV-SupT1	3	1	1	2	1	1
HIV-293T	4	4	1	0	0	0
HIV-Jurkat	2	1	1	2	3	0
HIV-IMR90	9	0	0	0	0	0
HIV-PBMC	3	2	1	2	1	0
L1-Hela	9	0	0	0	0	0
L1-Hela/HCT	9	0	0	0	0	0
MLV-HeLa-S	0	1	2	2	2	2
MLV-HeLa-NS	2	4	3	0	0	0
SFV-CD34+	9	0	0	0	0	0
SFV-Fibro	9	0	0	0	0	0
SB-Hela	9	0	0	0	0	0
SB-Huh-7	9	0	0	0	0	0

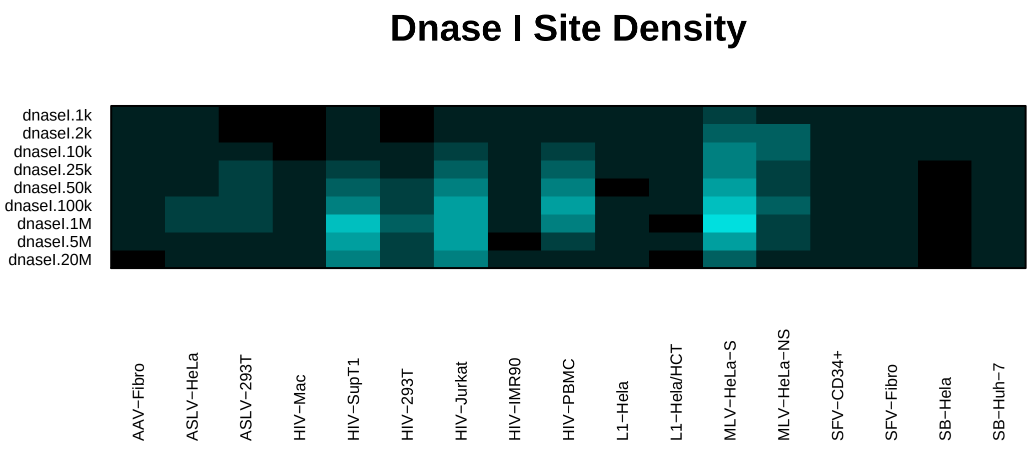

7.4 GC Content and CpG Islands

The distribution of ROC curve increments is given in this table:

	≤ 0.01	(0.01,0.02]	(0.02,0.03]	(0.03,0.04]	(0.04,0.05]	>0.05
AAV-Fibro	18	0	0	0	0	0
ASLV-HeLa	11	7	0	0	0	0
ASLV-293T	18	0	0	0	0	0
HIV-Mac	18	0	0	0	0	0
HIV-SupT1	16	2	0	0	0	0
HIV-293T	16	2	0	0	0	0
HIV-Jurkat	13	4	1	0	0	0
HIV-IMR90	18	0	0	0	0	0
HIV-PBMC	15	2	1	0	0	0
L1-Hela	18	0	0	0	0	0
L1-Hela/HCT	17	1	0	0	0	0
MLV-HeLa-S	8	4	2	3	1	0
MLV-HeLa-NS	12	3	3	0	0	0
SFV-CD34+	17	1	0	0	0	0
SFV-Fibro	18	0	0	0	0	0
SB-Hela	18	0	0	0	0	0
SB-Huh-7	18	0	0	0	0	0

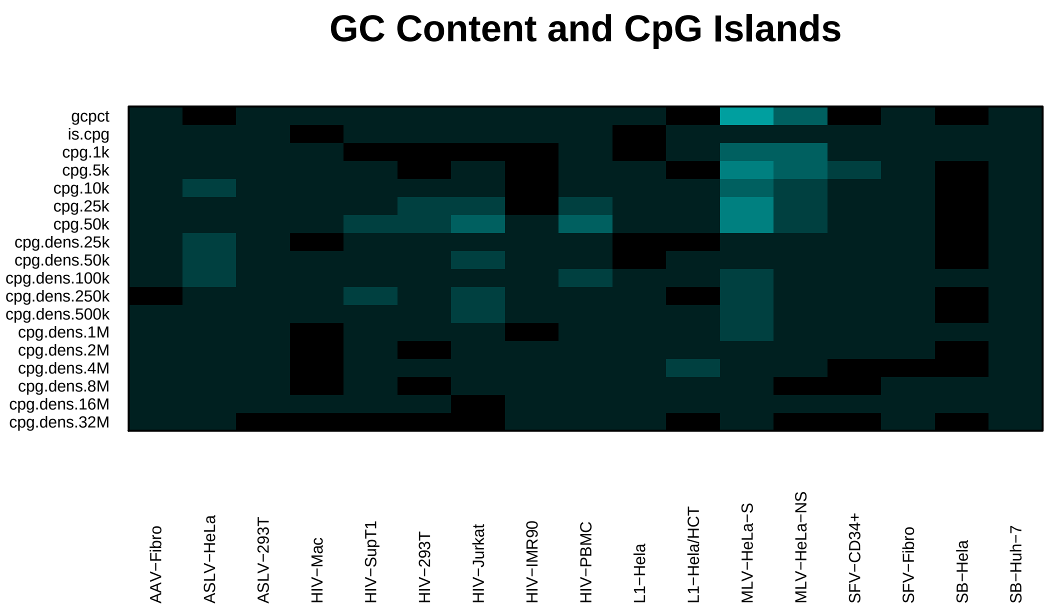

7.5 Transcription Start/Stop Features

The distribution of ROC curve increments is given in this table:

	≤ 0.01	(0.01,0.02]	(0.02,0.03]	(0.03,0.04]	(0.04,0.05]	>0.05
AAV-Fibro	20	0	0	0	0	0
ASLV-HeLa	17	3	0	0	0	0
ASLV-293T	19	1	0	0	0	0
HIV-Mac	14	4	2	0	0	0
HIV-SupT1	13	1	1	2	2	1
HIV-293T	14	1	5	0	0	0
HIV-Jurkat	11	2	1	0	2	4
HIV-IMR90	14	4	2	0	0	0
HIV-PBMC	11	2	1	1	4	1
L1-Hela	20	0	0	0	0	0
L1-Hela/HCT	17	2	0	1	0	0
MLV-HeLa-S	8	4	3	3	2	0
MLV-HeLa-NS	13	7	0	0	0	0
SFV-CD34+	20	0	0	0	0	0
SFV-Fibro	20	0	0	0	0	0
SB-Hela	20	0	0	0	0	0
SB-Huh-7	20	0	0	0	0	0

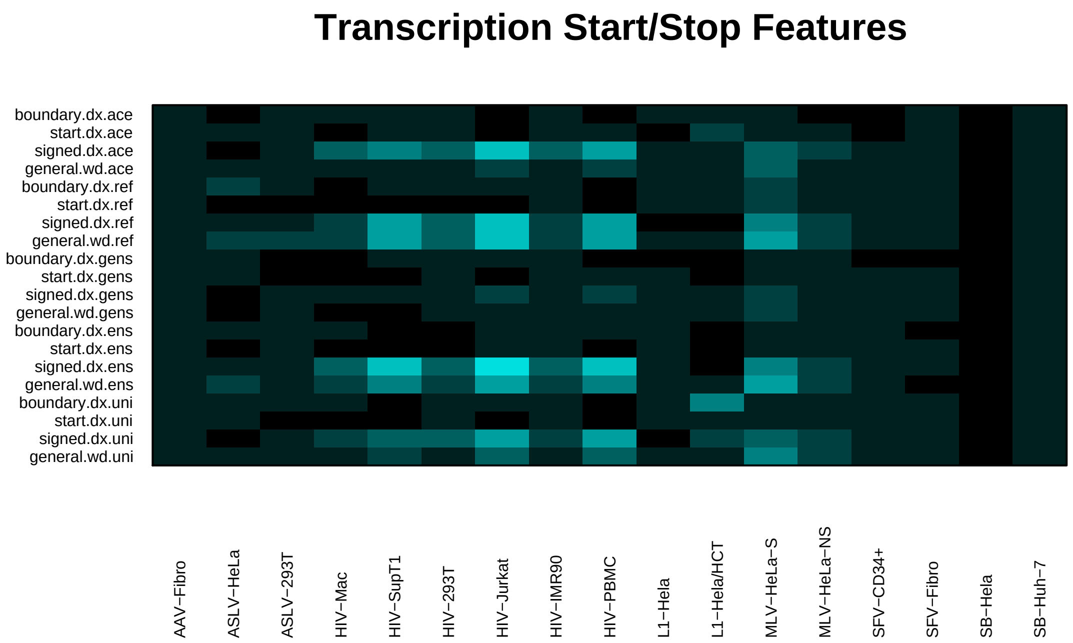

7.6 TRANSFAC scores

The distribution of ROC curve increments is given in this table:

	≤ 0.01	(0.01,0.02]	(0.02,0.03]	(0.03,0.04]	(0.04,0.05]	>0.05
AAV-Fibro	109	1	0	0	0	0
ASLV-HeLa	103	7	0	0	0	0
ASLV-293T	110	0	0	0	0	0
HIV-Mac	110	0	0	0	0	0
HIV-SupT1	105	5	0	0	0	0
HIV-293T	110	0	0	0	0	0
HIV-Jurkat	110	0	0	0	0	0
HIV-IMR90	110	0	0	0	0	0
HIV-PBMC	110	0	0	0	0	0
L1-Hela	110	0	0	0	0	0
L1-Hela/HCT	110	0	0	0	0	0
MLV-HeLa-S	86	19	5	0	0	0
MLV-HeLa-NS	104	6	0	0	0	0
SFV-CD34+	110	0	0	0	0	0
SFV-Fibro	110	0	0	0	0	0
SB-Hela	110	0	0	0	0	0
SB-Huh-7	110	0	0	0	0	0

TRANSFAC scores

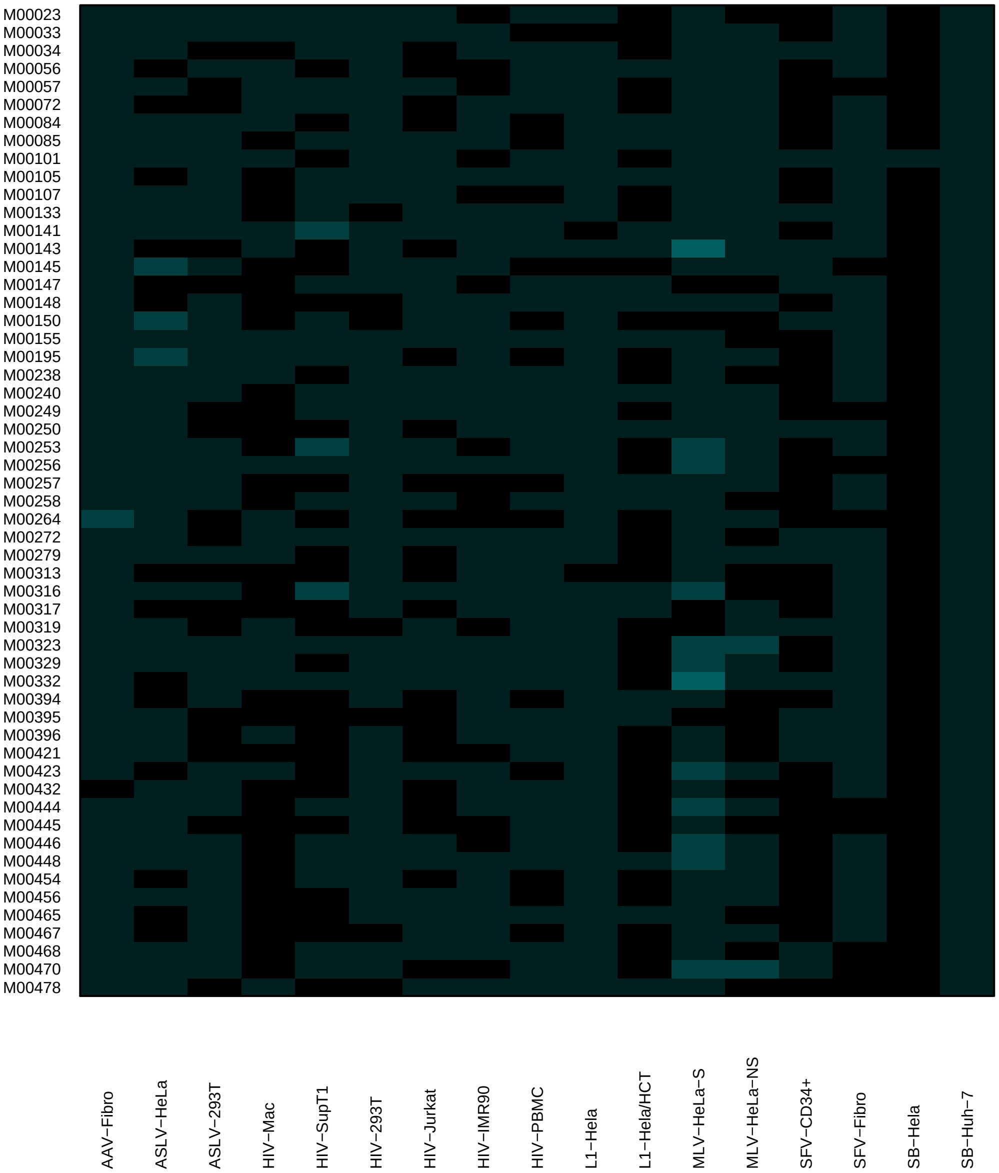

TRANSFAC scores (continued)

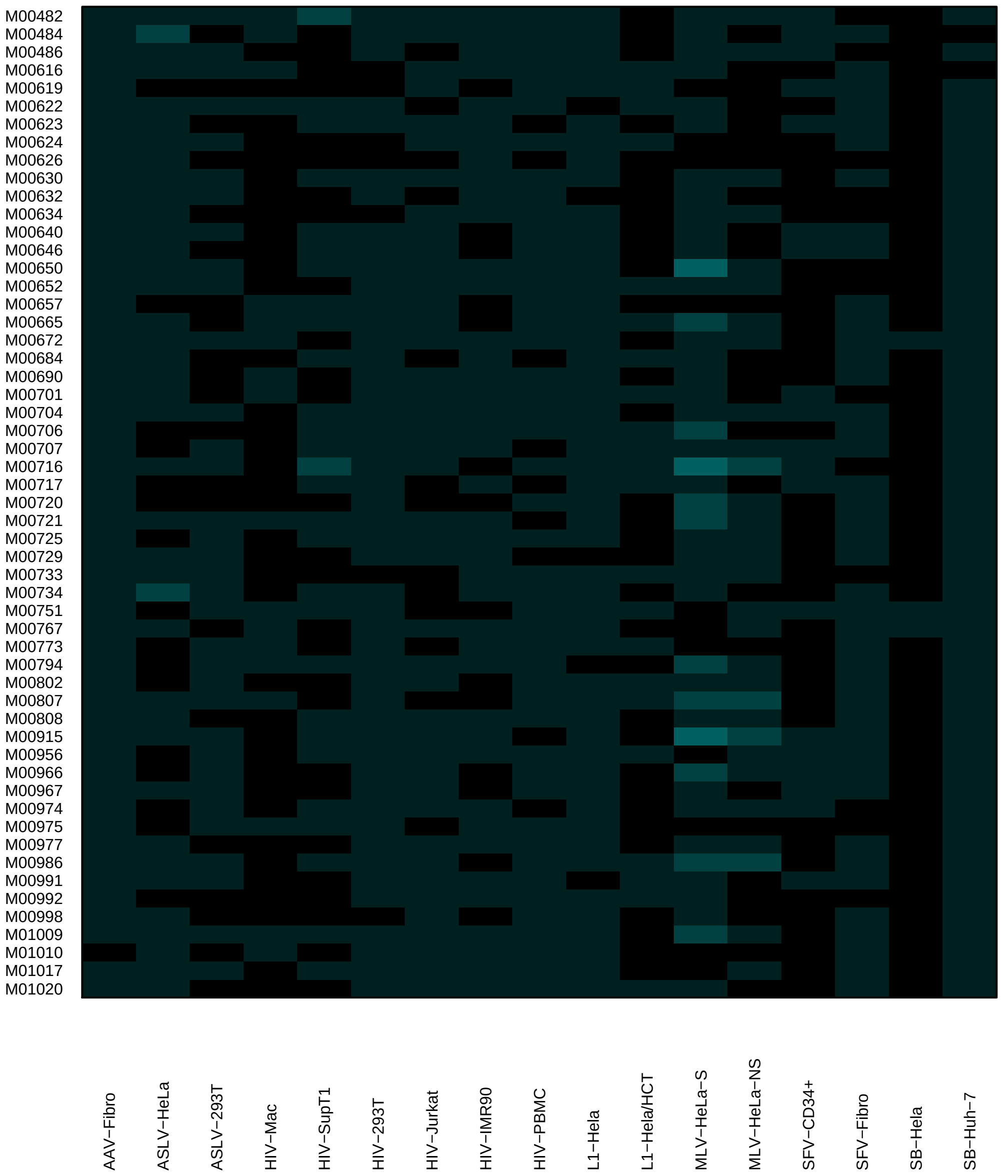

As is evident from inspection of the above graphs, there are many features that offer at least modest improvement of area under the ROC curve beyond what `score.20` offers. Plausibly, combining several of the features would lead

to a substantial improvement in the ability to predict integration preferences.

8 Combined Effects of Features

While some patterns have emerged from the results above, it is unclear how the features will behave in combination. Logistic or conditional logit regression methods can be used to fit selected features, but fitting a large number of features with datasets of practical size leads to results that are uninterpretable even when the computational difficulties can be resolved. Consideration of chosen subsets of features is practical, but given the number of features to be explored there are more than 10^{70} such combinations. The possible combinations of features is sometimes referred to as the *model space*. Methods are available that allow for searching over the model space to select models that fit well according to model selection criteria such as the Bayesian posterior probability or its approximation the Bayes Information Criterion (BIC) which balances the likelihood under a particular model against the number of variables it uses. By either collecting models sampled according to their posterior probabilities [George and McCulloch, 1993] or by collecting fewer models with the highest posterior probabilites [Raftery et al., 2005], summaries of the behavior of the different features can be obtained that integrate across the model space.

For example, each model has a Bayesian posterior probability associated with it, so summaries of the impact of differing classes of features can be obtained by summation of the posteriors for models that include one or more members of a given class. Classes with posterior probabilities much below 1.0 for a particular integration complex can be dismissed as unimportant.

For those classes that have high posterior probability, the posterior probabilities of features within that class and the posterior means of their regression coefficients can highlight important individual features.

Another approach is given by machine learning procedures, such as the random Forest™algorithm [Breiman, 2001], that have a high degree of flexibility in forming classification rules from large collections of features.

8.1 Regression via Bayes Model Averaging

The regression modelling was carried out using the R package `BMA`[Raftery et al., 2005] using `bic.glm` (for random genomic controls) and a version of `bic.surv` (for matched random controls) slightly modified to perform conditional logit analysis. When `bic.glm` or `bic.surv` is used with 30 or fewer regressor variables it uses a branch-and-bound strategy [Lawless and Singhal, 1978] to prune models of low posterior probability out of the more than one billion possible models back to a manageable number of models. Each of these models is fitted, and the posterior probability, coefficients, and standard errors of each model are saved. When used with 31 or more variables it starts by fitting all variables then performing a backward deletion to prune down to thirty variables, then it applies the other procedures. With more than 200 variables here, this strategy will fail.

The computing time needed would be excessive, and the elimination procedure may prune out variables that would do well in smaller models. To cope with this a Bayesian linear regression procedure that performs a stochastic search in the model space is used to order the variables according to their posterior probability under the linear regression heuristic. The best thirty of these are used in `bic.glm` or `bic.surv`. In addition, the consistent effects of many gene or expression density features led us to perform a data reduction in which the first principal component of the ranks of the gene or expression density variables was extracted, and this variable (`pc1`) was added to the list of candidate variables so that weak effects distributed over many variables couold be included in a parsimonious manner. The initial linear model stochastic search conditioned on inclusion of both variables. In order to obtain comparable results across the full collection of features, a transformation was applied to all variables (except the gene or exon features which are coded with zero or one indicators); the rank of each feature are rescaled to range from -1 to 1. Thus, the quartiles are -0.50 and 0.50, a one unit difference apart. A coefficient of 0.50 implies that one unit difference would change the log-odds of integration by 0.50 (or increase the odds by about 65 percent).

The overall effect of adding variables to `score.20` is summarized in the following table. K-fold cross-validation (with $k = 10$) is used to obtain honest estimates of the area under the ROC curve that would be obtained with fresh observations; each training sample is used to select the variables to be included and to fit the model, while the fitted values are computed for the corresponding test set and the ROC curve area is computed using all test sets together.

	score.20	BMA.fit	Improvement
AAV-Fibro	0.64	0.65	0.02
ASLV-HeLa	0.72	0.74	0.01
ASLV-293T	0.77	0.79	0.02
HIV-Mac	0.83	0.88	0.05
HIV-SupT1	0.81	0.92	0.10
HIV-293T	0.84	0.90	0.06
HIV-Jurkat	0.83	0.92	0.09
HIV-IMR90	0.82	0.85	0.03
HIV-PBMC	0.83	0.91	0.08
L1-Hela	0.99	0.99	0.00
L1-Hela/HCT	0.82	0.80	−0.02
MLV-HeLa-S	0.81	0.90	0.09
MLV-HeLa-NS	0.79	0.84	0.05
SFV-CD34+	0.80	0.83	0.03
SFV-Fibro	0.78	0.79	0.01
SB-Hela	0.84	0.83	−0.01
SB-Huh-7	0.99	0.99	−0.00

The HIV and MLV integration complexes show substantial improvement with the additional variables. In the other integration complexes, the improvement is more modest. In a few cases there is negative 'improvement'; this may

be a consequence of optimizing the log-likelihood criterion and then testing with the ROC curve criterion.

To determine the similarity of scores developed using BMA for the different integration complexes, a sample of random genomic sites is scored using each of the rules and the correlations of those scores are displayed in false color in the following figure. Green corresponds to negative correlations (meaning that when one integration complex is favored the other integration complex tends to be disfavored) and red corresponds to positive correlations (both complexes tend to be favored or disfavored in tandem). The integration complexes are subjected to hierarchical clustering of the correlations, which is shown on the figure, and ordered according to cluster membership.

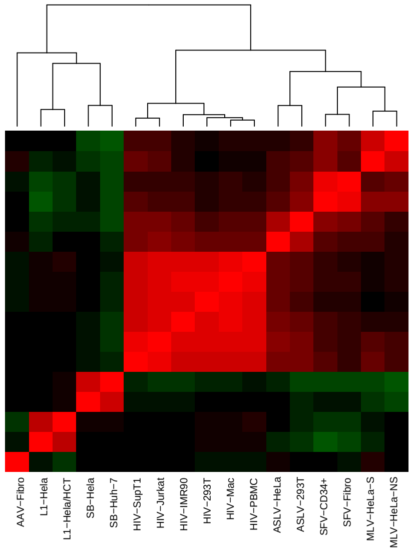

Note that the ordering of the rows and columns may have changed compared to the earlier image that was based only on `score.20`. So, it is necessary to study the labels to make comparisons. Overall, the image is very similar to that seen earlier which was based only on `score.20`. Given the strong effects of that variable this is not a surprise. The correlations within the HIV group of integration complexes now are all slightly reduced compared to the earlier figure. The ASLV complexes now are slightly more correlated with the MLV complexes and the negative correlations of "AAV-Fibro" complex with the HIV complexes are now essentially nil.

The posterior probability associated with each class of features is presented in the table below.

	gene.exon	gene.density	cpg	dnase	juxtapos	transfac	score.20
AAV-Fibro	0.03	1.00	0.08	0.00	1.00	1.00	1.00
ASLV-HeLa	0.07	1.00	0.99	0.00	1.00	1.00	1.00
ASLV-293T	0.10	1.00	1.00	1.00	0.00	0.98	1.00
HIV-Mac	1.00	1.00	1.00	1.00	0.00	1.00	1.00
HIV-SupT1	1.00	1.00	1.00	1.00	0.00	1.00	1.00
HIV-293T	1.00	1.00	1.00	1.00	0.36	1.00	1.00
HIV-Jurkat	1.00	1.00	1.00	1.00	1.00	0.98	1.00
HIV-IMR90	1.00	1.00	1.00	0.92	0.00	0.99	1.00
HIV-PBMC	1.00	1.00	1.00	1.00	1.00	1.00	1.00
L1-Hela	0.00	0.00	0.12	0.00	0.81	0.00	1.00
L1-Hela/HCT	0.13	0.93	0.02	0.28	0.88	0.13	1.00
MLV-HeLa-S	0.00	1.00	1.00	1.00	0.99	0.00	1.00
MLV-HeLa-NS	0.51	1.00	1.00	1.00	1.00	1.00	1.00
SFV-CD34+	1.00	1.00	1.00	1.00	1.00	0.89	1.00
SFV-Fibro	1.00	1.00	1.00	1.00	1.00	1.00	1.00
SB-Hela	0.03	0.00	1.00	0.00	0.11	0.74	1.00
SB-Huh-7	0.29	0.96	0.67	0.36	0.00	0.44	1.00

The only class that shows high posterior probability in every integration complex is the single variable `score.20` – the score based on the positional weight matrix of the 20 base pairs flanking the integration site.

The integration complexes "SB-Huh-7" and "L1-Hela" show low posterior probabilities for almost all of the other classes. However, this might be expected since `score.20` is an accurate predictor of integration by itself for these complexes.

The integration complexes "SB-Hela" and "L1-Hela/HCT" that showed negative "improvement" of the model with multiple variables over that with `score.20` alone (in terms the the area under the ROC curve) only show high posterior probability in one or two classes besides the `score.20` class.

The class `gene.density` has high posterior probability in all integration complexes but "SB-Hela and "L1-Hela". The class `gene.exon` has low posterior probability in about half of the integration complexes, while very high posterior probability is shown in at least a majority of the integration complexes. This is also true of the `juxtapos` class. The other classes show high posterior probability for the majority of integration complexes.

Another view of the impact of each class of features is obtained by examining the influence each feature has on the log-odds of integration. The predicted log-odds of integration for location (i, j, k) are proportional to

$$\log \phi_{ijk} = X_{ijk}\beta$$

where X_{ijk} is the vector of feature values for that location and β is the vector of regression coefficients corresponding to those features. This can be decomposed by partitioning X and β rendering

$$\log \phi_{ijk} = X_{ijk}^{(gene.exon)}\beta_{(gene.exon)} + \cdots + X_{ijk}^{(transfac)}\beta_{(transfac)}$$

The contributions, $X_{ijk}^{(r)}\beta_{(r)}$, can be evaluated separately. In the following table, the variance of the contribution from each feature type is estimated using a sample of control locations drawn at random from the genome for each of the 17 integration complexes.

	score.20	cpg	dnase	gene.density	gene.exon	juxtapos	transfac
AAV-Fibro	0.26	0.00	0.00	0.23	0.00	0.17	0.06
ASLV-HeLa	0.80	0.06	0.00	0.38	0.00	0.12	0.08
ASLV-293T	1.23	0.09	0.05	0.07	0.00	0.00	0.02
HIV-Mac	2.51	0.29	0.30	0.27	0.14	0.00	0.09
HIV-SupT1	2.19	1.08	0.61	0.77	0.20	0.00	0.22
HIV-293T	2.78	0.59	0.22	0.37	0.10	0.00	0.04
HIV-Jurkat	2.56	0.77	0.09	1.34	0.19	0.09	0.03
HIV-IMR90	2.04	0.24	0.03	0.45	0.09	0.00	0.06
HIV-PBMC	2.37	0.44	0.50	0.44	0.20	0.10	0.12
L1-Hela	762.45	0.01	0.00	0.00	0.00	0.39	0.00
L1-Hela/HCT	2.65	0.00	0.03	0.16	0.00	0.25	0.00
MLV-HeLa-S	1.44	0.49	0.47	0.22	0.00	0.04	0.00
MLV-HeLa-NS	1.62	0.17	0.12	0.17	0.00	0.06	0.07
SFV-CD34+	1.60	0.19	0.10	0.10	0.03	0.03	0.01
SFV-Fibro	1.48	0.05	0.03	0.04	0.04	0.03	0.04
SB-Hela	2.95	0.29	0.00	0.00	0.00	0.00	0.06
SB-Huh-7	226.13	0.06	0.01	0.22	0.02	0.00	0.01

As can be seen the largest variance for each integration complex is that due to `score.20`, while `gene.density`, `cpg`, and `dnase` all had at least one component that had a variance of at least 0.50. Note that with a variance of 0.5 that two locations differing only on that component and by just one standard deviation would have relative odds of $\exp(\sqrt{2}) \approx 2.03$ — a moderate difference in odds.

We turn now to a consideration of the individual features in each class. The regressor variables used were transformed by taking ranks of the values in each data set and then scaling them to have unit variance. This allows the magnitudes of the regression coefficients to be compared as a means of assessing the importance of each variable in integration targetting. For `score.20` the coefficients are all large.; the smallest value is 0.88 for AAV-Fibro and the next smallest is 1.55 for ASLV-HeLa.

In what follows, the other regression coefficient posterior means are represented graphically in false color with increasing intensity of green indicating more negative values and increasing intensity of red indicating more positive values. The scale is shown in the follow figure. A small number of values exceed the range and are shown in the brightest colors.

Color Map Key for BMA

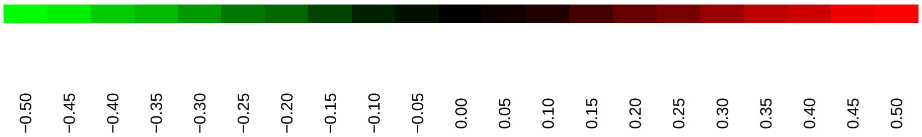

8.1.1 Gene or Exon

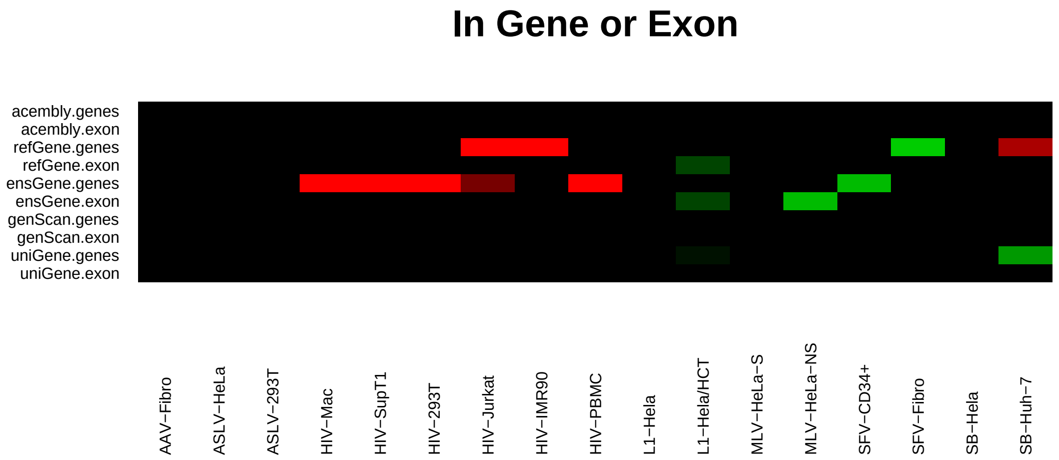

8.1.2 Gene or Expression Density

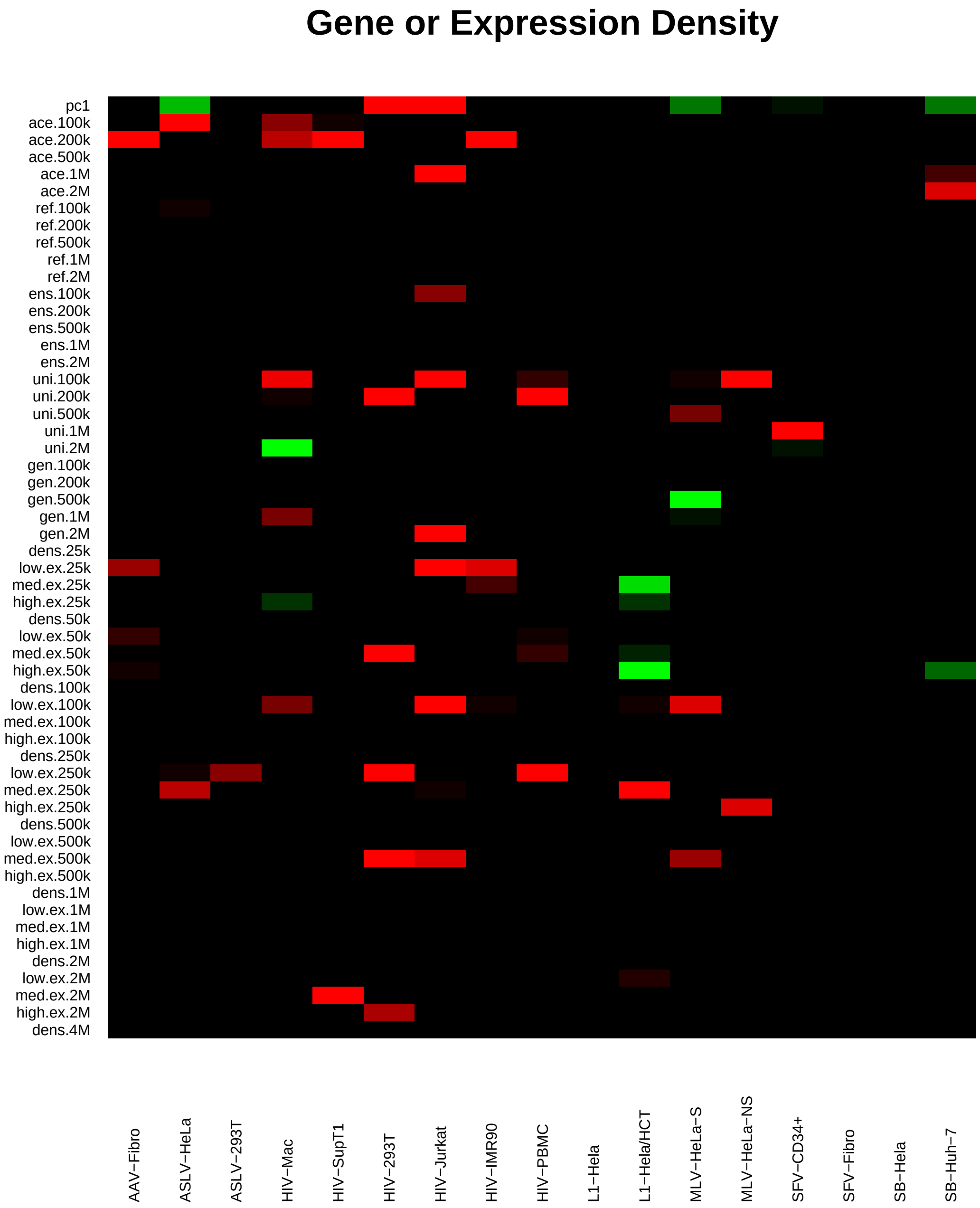

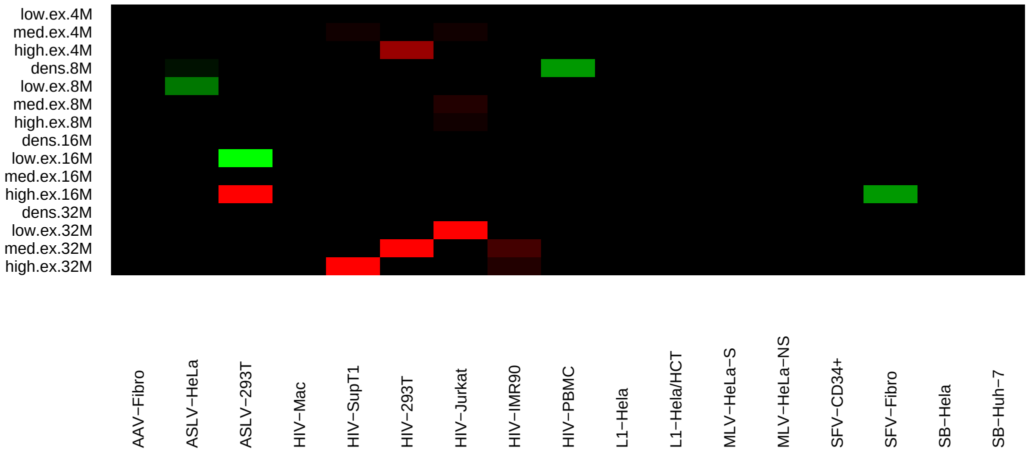
low.ex.4M
med.ex.4M
high.ex.4M
dens.8M
low.ex.8M
med.ex.8M
high.ex.8M
dens.16M
low.ex.16M
med.ex.16M
high.ex.16M
dens.32M
low.ex.32M
med.ex.32M
high.ex.32M
AAV−Fibro
ASLV−HeLa
ASLV−293T
HIV−Mac
HIV−SupT1
HIV−293T
HIV−Jurkat
HIV−IMR90
HIV−PBMC
L1−Hela
L1−Hela/HCT
MLV−HeLa−S
MLV−HeLa−NS
SFV−CD34+
SFV−Fibro
SB−Hela
SB−Huh−7

8.1.3 Dnase I Site Density

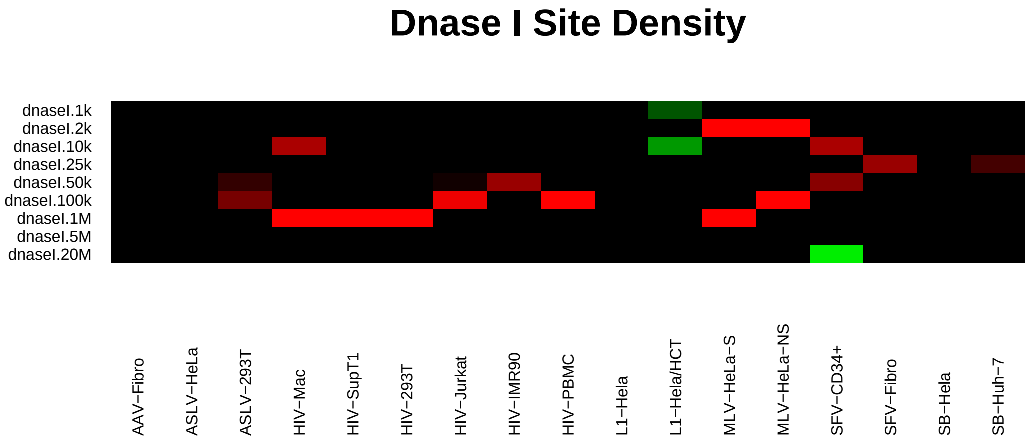

8.1.4 GC Content and CpG Islands

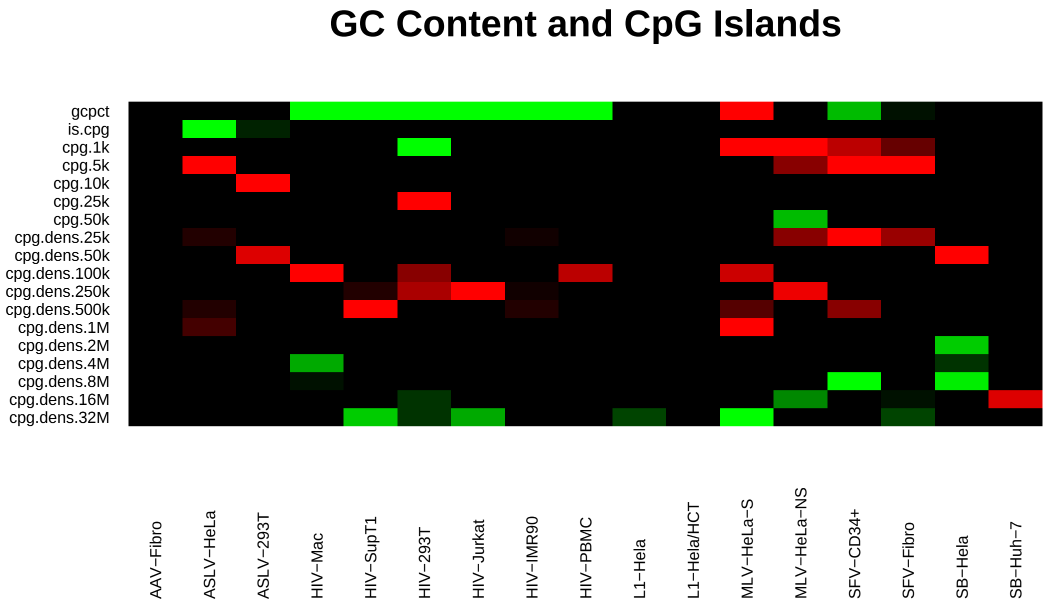

8.1.5 Transcription Start/Stop Features

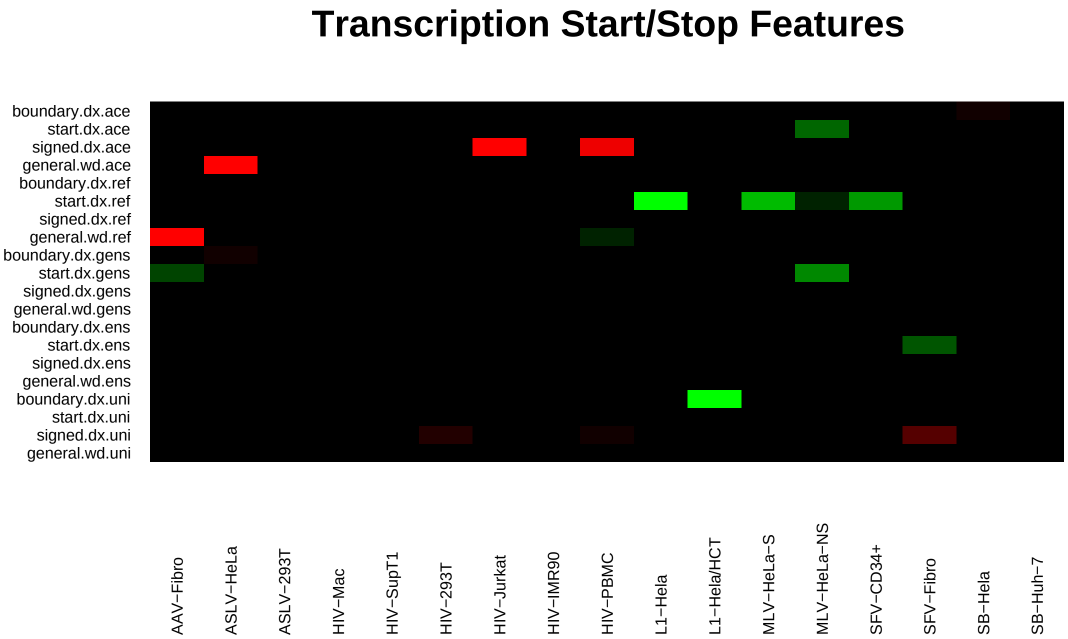

8.1.6 TRANSFAC scores

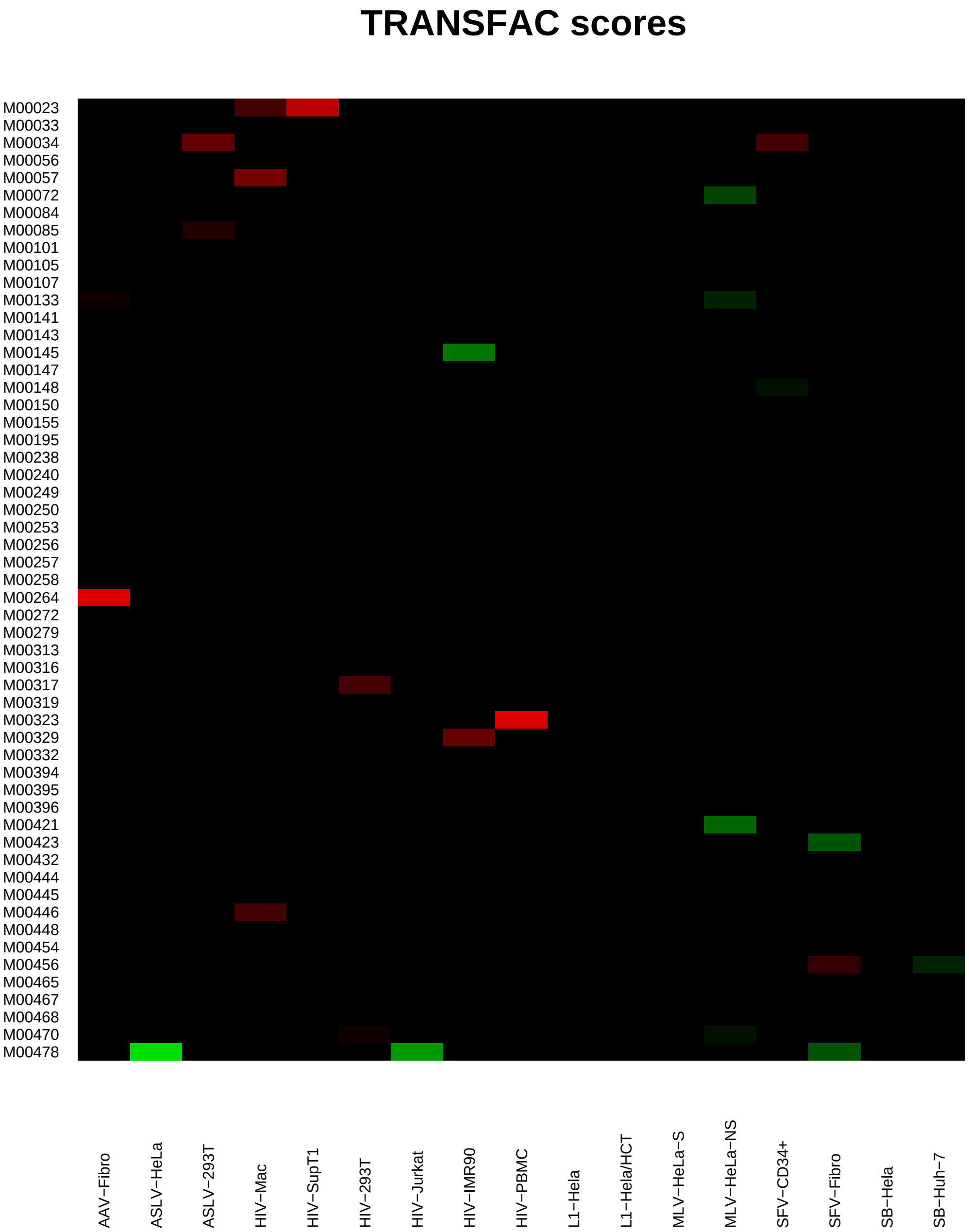

TRANSFAC scores (continued)

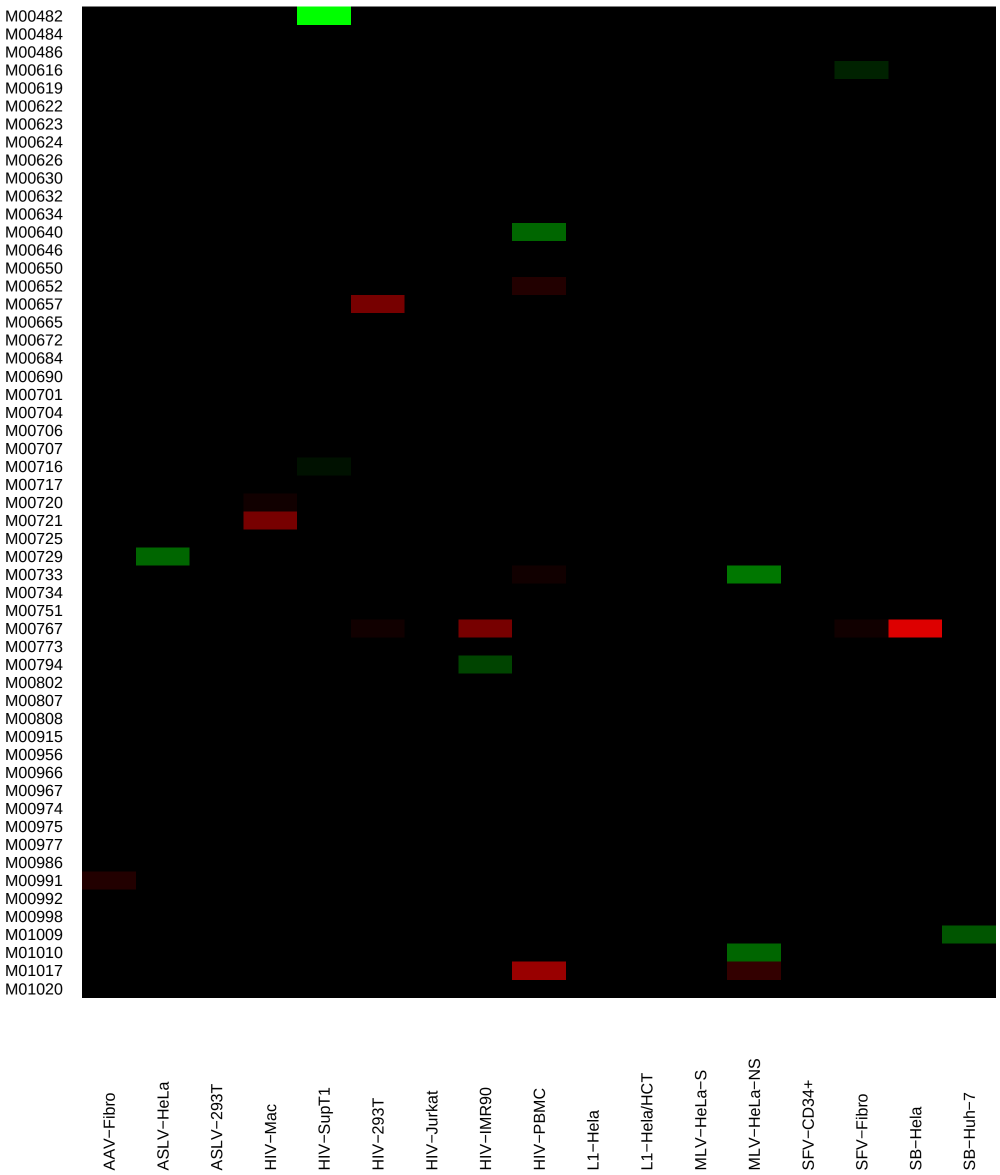

As HIV complexes showed the most improvement, it is probably not surprising that they also showed many stronger effects. Many gene or expression density variables showed positive effects for HIV and few showed negative ef-

fects, although the window widths and annotation that showed the effects varied for the different HIV complexes. To a lesser extent, this was also true of MLV. Also, being in a refSeq or Ensembl gene showed a positive effect for each HIV complex.

Having nearby (±500 bases) dnase I sites was not influential although having dnase I sites in a broader region was for both HIV and MLV.

The negative effect for `gcpct` on HIV integration is a surprise; in earlier displays this effect was nil or very small. Evidently, a suppressor variable relationship underlies this. CpG densities show some positive and some negative effects.

The features that relate to the transcription start sites show few effects. In particular, the `signed.dx` features that all showed positive associations with MLV and HIV integration are mostly nil in this context.

The TRANSFAC scores are mostly nil. Those that are not nil are roughly evenly divided between positive and negative effects and show no obvious pattern of concordance.

8.2 Fitting via RandomForests ™

The random Forest ™algorithm as implemented in the R package `randomForest`[Liaw and Wiener, 2002] was used to fit the data from each integration site. This was done separately using just the `score.20` and `pc1` variables, adding just the TRANSFAC scores to those two, adding the other features to those two, and using all features together.

Each fit produces a 'vote' on the category (integration site vs control site) that can be used to calculate the area under the ROC curve. The fit for each site is done by using a collection of training samples that do not include the site in question (known as "out-of-bag" prediction), so overfitting is not an issue.

The table below shows the area under the ROC curve for `score.20` alone and for each of the votes from the randomForest classifiers: `and pc1` which uses the principal component score for gene density as well as `score.20`,`and others` which uses `score.20` and `pc1` and all features besides the TRANSFAC scores, `and PWM` which uses the TRANSFAC scores, and `All Features`.

	score.20	20 and pc1	and others	and PWM	All Features
AAV-Fibro	0.64	0.54	0.65	0.60	0.63
ASLV-HeLa	0.72	0.68	0.66	0.65	0.65
ASLV-293T	0.77	0.71	0.73	0.71	0.71
HIV-Mac	0.83	0.80	0.84	0.82	0.84
HIV-SupT1	0.81	0.83	0.88	0.85	0.88
HIV-293T	0.84	0.82	0.88	0.86	0.88
HIV-Jurkat	0.83	0.84	0.90	0.87	0.90
HIV-IMR90	0.82	0.78	0.82	0.80	0.81
HIV-PBMC	0.83	0.81	0.90	0.84	0.89
L1-Hela	0.99	0.96	0.98	0.93	0.96
L1-Hela/HCT	0.82	0.71	0.72	0.71	0.72
MLV-HeLa-S	0.81	0.83	0.87	0.84	0.86
MLV-HeLa-NS	0.79	0.76	0.82	0.79	0.81
SFV-CD34+	0.80	0.73	0.81	0.77	0.80
SFV-Fibro	0.78	0.70	0.77	0.72	0.75
SB-Hela	0.84	0.80	0.90	0.80	0.82
SB-Huh-7	0.99	0.98	0.98	0.98	0.98

In many instances, the randomForest vote produces poorer results than `score.20` all alone. This is probably a reflection of the limited ability of randomForest to capture weak monotone effects in datasets of the size used here. Usually, the best randomForest is based on `score.20`, `pc1`, and the "other" features alone. While the TRANSFAC scores sometimes yield higher ROC curve areas than `score.20` and `pc1` alone, they never do better than the "other" features. "All Features" is usually a close second to "`and others`" suggesting that the TRANSFAC features add no useful information. Further, these results are almost always less than those for the Bayes Model Averaging predictions — sometimes by a substantial margin.

Finally, we combined the randomForest results with the Bayes Model Average results by using the votes and the BMA prediction as regressors in a conditional logit model (for 'match'ed controls) or logistic regression model (for 'random' controls). Using the fitted values, the ROC curve area is again computed. This is done without further crossvalidation. The results shown here compare the Bayes Model Average predictions to the combination (`combo`). The p-value is based on the difference in ROC curve areas[DeLong et al., 1988].

	BMA	combo	p.value
AAV-Fibro	0.65	0.69	0.0002
ASLV-HeLa	0.74	0.73	0.5194
ASLV-293T	0.79	0.79	0.2389
HIV-Mac	0.88	0.89	0.0005
HIV-SupT1	0.92	0.92	0.2117
HIV-293T	0.90	0.91	0.0002
HIV-Jurkat	0.92	0.93	0.0005
HIV-IMR90	0.85	0.86	0.0059
HIV-PBMC	0.91	0.92	0.0001
L1-Hela	0.99	0.99	0.0058
L1-Hela/HCT	0.80	0.83	0.0503
MLV-HeLa-S	0.90	0.91	0.1701
MLV-HeLa-NS	0.84	0.85	0.0002
SFV-CD34+	0.83	0.84	0.0000
SFV-Fibro	0.79	0.80	0.0012
SB-Hela	0.82	0.89	0.0000
SB-Huh-7	0.99	0.99	0.0005

Rather small increases in ROC curve area are seen for most integration complexes. The most substantial is seen for the "SB-Hela" complex. Further analysis (not shown here) suggests that this is due to effects that manifest when `score.20` is not at its highest levels. This type of effect would be represented as an interaction effect in a regression model. The randomForest algorithm picks up such interaction effects automatically, while the regression models used here did not allow for them. Further studies may attempt to the fit regression models with interaction effects. However, the mostly modest increases seen in ROC curve areas when fitting both randomForest votes and BMA predictions suggests that interaction effects are usually minor.

References

[Bishop et al., 1975] Bishop, Y. M. M., Fienberg, S. E., and Holland, P. W. (1975). *Discrete Multivariate Analyses: Theory and Practice.* MIT Press.

[Breiman, 2001] Breiman, L. (2001). Random forests. *Machine Learning*, 45:5–32.

[Breslow, 1996] Breslow, N. E. (1996). Statistics in epidemiology: the case-control study. *J Am Stat Assoc*, 91(433):14–28.

[DeLong et al., 1988] DeLong, E. R., DeLong, D. M., and Clarke-Pearson, D. L. (1988). Comparing the areas under two or more correlated receiver operating characteristic curves: A nonparametric approach. *Biometrics*, 44:837–845.

[George and McCulloch, 1993] George, E. I. and McCulloch, R. E. (1993). Variable selection via Gibbs sampling. *Journal of the American Statistical Association*, 88:881–889.

[Jones and Vines, 1998] Jones, M. C. and Vines, S. K. (1998). Choosing the smoothing parameter for unordered multinomial data. *Test (Madrid)*, 7:413–426.

[Lawless and Singhal, 1978] Lawless, J. F. and Singhal, K. (1978). Efficient screening of nonnormal regression models. *Biometrics*, 34:318–327.

[Liaw and Wiener, 2002] Liaw, A. and Wiener, M. (2002). Classification and regression by randomforest. *R News*, 2(3):18–22.

[McCullagh and Nelder, 1999] McCullagh, P. and Nelder, J. A. (1999). *Generalized Linear Models.* Chapman & Hall Ltd.

[Raftery et al., 2005] Raftery, A., Hoeting, J., Volinsky, C., and Painter, I. (2005). *BMA: Bayesian Model Averaging.* R package version 3.01.
