# Supplementary material for: Selection of Target Sites for Mobile DNA Integration in the Human Genome
Source: PLoS Comput Biol. 2006 Nov 24;2(11):e157. doi: 10.1371/journal.pcbi.0020157 (PMC1664696; doi:10.1371/journal.pcbi.0020157)
Supplement: Text S3 — (43 KB DOC) [file pcbi.0020157.sd003.doc]

**Clustering Transcription factor PWMs**

We wished to analyze the effects of TransFac PWMs on integration using the ROC curve approach. However, there is consdiderable redundancy in the TransFac database. Thus there is the risk of over-emphasizing particular PWMs that are simply redundant representations of a single binding site. For that reason, the TransFac motifs were clustered, and representative PWMs from each cluster were analyzed. This aspect of the analysis is desribed below.

*Pair-wise similarity computation.* Each PWM *X* is a 4 by *k* matrix for *k*-length binding site, where X_ui_ is the proportion of base *u* at position *i,* such that (Stormo, 2000). We compute the dissimilarity or distance between position *i* of PWM *X* and position *j* of PWM *Y* using relative entropy (Durbin et al., 1998). For two identical positions this value is 0 and the more dissimilar the positions, the higher the RE value. However, as defined, this is an asymmetric measure and in practice we take the average of *R_ij_* and *R_ji_* as the distance between the two positions. Notice that according to this measure, for two positions at which the base pairs are distributed according to the background probability (say, equi-probable), their RE value will be 0, even though individually these positions are not informative. Let *R_ir_* be the RE-value between column *i* and background probability distribution of bases. *R_jr_* is defined similarly. We define the similarity between column *i* and column *j,* *.* We first compute the *S_ij_* for every pair of columns for all PWMs in the TRANSFAC database. These values are normally distributed with mean μ and standard deviation σ. The sum of *k* such S-values is also normally distributed with mean *μ_k_=μk*, and standard deviation *σ_k_=*σ√k. To compute the similarity between *k* consecutive columns of two PWMs, we sum up the *k S*-values for aligned column pairs and transform this value to a z-score = (S - *μ_k_*)/*σ_k_*, which makes the scores for different values of *k* comparable. Next, for every PWM-pair and for every alignment offset with a minimum of 6 base overlap between the PWMs (ie., *k* ≥ 6), we compute the similarity z-score (“z-value”). Using the empirical distribution of z-values for all alignments of all PWM pairs, we convert each individual z-value into a p-value, ie., the probability of observing the z-value or higher in the background distribution; we call this the pz-value. Finally, to compute the similarity between two PWMs *X* and *Y* while allowing for the possibility that two related PWMs may be slightly shifted in positions, we slide the PWMs relative to each other such that at least 6 positions are aligned. For each such offset we compute the *pz*-value. Let *mpz* be the minimum *pz*-value over all offsets. Notice that the longer PWM pairs have a greater number of possible offsets and thus tend to achieve a low *mpz*-value. To correct for this effect, we compute the significance of the observed *mpz*-value as the random expectation of observing the mpz-value for *K* trials where *K* is the number of offsets. That is,

.

*Clustering PWMs based on the P-values.* Given a p-value threshold (we use 0.02), all PWMs can be represented as a network where PWMs correspond to the nodes and two nodes are connected if their similarity p-value is below the threshold. We then compute the so-called bi-connected component in this graph. A bi-connected component is a connected component of the graph that remains connected if any of the nodes are removed. Each bi-connected component corresponds to a cluster. In other words if two PWMs belong to same cluster, they must have at least two independent lines of evidence that they are related (ie., paths in the graph). Each cluster thus obtained represents a family of PWMs with similar DNA binding specificity. We selected the median of each cluster as the cluster representative. Out of 546 PWMs, 495 were grouped into 59 clusters, and with 51 singletons, this procedure resulted in 110 representative PWMs (Table 1).

Table 1. 110 representative positional weight matrices from TRANSFAC.

| **TRANSFAC PWM ID** | **Factor Name** |
| --- | --- |
| M00143 | BSAP |
| M00975 | RFX |
| M00650 | MTF-1 |
| M00966 | VDR,_CAR,_PXR |
| M00665 | Sp3 |
| M00634 | GCM |
| M00249 | CHOP:C/EBPalpha |
| M00991 | CDX |
| M00145 | Brn-2 |
| M01020 | TBX5 |
| M00329 | Pax-9 |
| M00396 | En-1 |
| M00701 | SMAD-3 |
| M00250 | Gfi-1 |
| M00456 | FAC1 |
| M00454 | MRF-2 |
| M00445 | Xvent-1 |
| M00423 | FOXJ2 |
| M00101 | CdxA |
| M01017 | PBX1 |
| M00056 | myogenin_/_NF-1 |
| M00470 | AP-2gamma |
| M00034 | p53 |
| M00240 | Nkx2-5 |
| M00448 | Zic1 |
| M00257 | RREB-1 |
| M00279 | MIF-1 |
| M00256 | NRSF |
| M00725 | HP1_site_factor |
| M00085 | ZID |
| M00992 | FOXP3 |
| M00622 | C/EBPgamma |
| M00258 | ISRE |
| M00967 | HNF4,_COUP |
| M00482 | PITX2 |
| M00084 | MZF1 |
| M01010 | HMGIY |
| M00623 | Crx |
| M00690 | AP-3 |
| M00808 | Pax |
| M00468 | AP-2rep |
| M00478 | Cdc5 |
| M00253 | cap |
| M00150 | Brachyury |
| M00272 | p53 |
| M00317 | Poly_A |
| M00619 | Alx-4 |
| M00986 | Churchill |
| M00316 | Imperfect_Hogness/Goldberg_BOX |
| M00720 | CAC-binding_protein |
| M01009 | HES1 |
| M00646 | LF-A1 |
| M00432 | TTF1 |
| M00332 | Whn |
| M00802 | Pit-1 |
| M00684 | XPF-1 |
| M00630 | FOXM1 |
| M00706 | TFII-I |
| M00264 | Staf |
| M00444 | VDR |
| M00395 | HOXA3 |
| M00033 | p300 |
| M00057 | COMP1 |
| M00394 | Msx-1 |
| M00998 | PBX |
| M00624 | DBP |
| M00486 | Pax-2 |
| M00767 | FXR_inverted_repeat_1 |
| M00446 | Spz1 |
| M00717 | Pax-8 |
| M00729 | Cdx-2 |
| M00915 | AP-2 |
| M00023 | Hox-1.3 |
| M00484 | Ncx |
| M00323 | Muscle_initiator_sequences-19 |
| M00807 | EGR |
| M00707 | TFIIA |
| M00657 | PTF1-beta |
| M00148 | SRY |
| M00421 | MEIS1B:HOXA9 |
| M00794 | TTF-1 |
| M00072 | CP2 |
| M00734 | CIZ |
| M00107 | E2 |
| M00467 | Roaz |
| M00319 | MEF-3 |
| M00632 | GATA-4 |
| M00465 | POU6F1 |
| M00141 | Lyf-1 |
| M00155 | ARP-1 |
| M00956 | AR |
| M00626 | RFX1_(EF-C) |
| M00616 | AFP1 |
| M00652 | Nrf-1 |
| M00977 | EBF |
| M00672 | TEF |
| M00974 | SMAD |
| M00721 | CACCC-binding_factor |
| M00640 | HOXA4 |
| M00238 | Barbie_Box |
| M00773 | MYB |
| M00313 | GEN_INI |
| M00133 | Tst-1 |
| M00733 | SMAD-4 |
| M00195 | Oct-1 |
| M00751 | AML1 |
| M00147 | HSF2 |
| M00105 | CDP_CR3 |
| M00716 | ZF5 |
| M00704 | TEF-1 |

**REFERENCES**

Durbin, R., S. Eddy, A. Krogh and G. Mitchison (1998). Biological Sequence Analysis. Cambridge, UK, Cabridge University Press.

Stormo, G. D. (2000). "DNA binding sites: representation and discovery." Bioinformatics **16**(1): 16-23.
